# The anti‐aging protein Klotho affects early postnatal myogenesis by downregulating Jmjd3 and the canonical Wnt pathway

**DOI:** 10.1096/fj.202101298R

**Published:** 2022-02-17

**Authors:** Cynthia M. McKee, Douglas J. Chapski, Michelle Wehling‐Henricks, Manuel Rosa‐Garrido, Makoto Kuro‐o, Thomas M. Vondriska, James G. Tidball

**Affiliations:** ^1^ Molecular, Cellular & Integrative Physiology Program University of California Los Angeles California USA; ^2^ Department of Anesthesiology and Perioperative Medicine David Geffen School of Medicine at UCLA Los Angeles California USA; ^3^ Department of Integrative Biology and Physiology University of California Los Angeles California USA; ^4^ Department of Biomedical Engineering, School of Medicine and School of Engineering University of Alabama at Birmingham Birmingham USA; ^5^ Division of Anti‐Aging Medicine, Center for Molecular Medicine Jichi Medical University Shimotsuke Japan; ^6^ Departments of Medicine David Geffen School of Medicine at UCLA Los Angeles California USA; ^7^ Departments of Physiology David Geffen School of Medicine at UCLA Los Angeles California USA; ^8^ Department of Pathology and Laboratory Medicine David Geffen School of Medicine at UCLA, University of California Los Angeles California USA

**Keywords:** development, myogenesis, skeletal muscle

## Abstract

Modulating the number of muscle stems cells, called satellite cells, during early postnatal development produces long‐term effects on muscle growth. We tested the hypothesis that high expression levels of the anti‐aging protein Klotho in early postnatal myogenesis increase satellite cell numbers by influencing the epigenetic regulation of genes that regulate myogenesis. Our findings show that elevated *klotho* expression caused a transient increase in satellite cell numbers and slowed muscle fiber growth, followed by a period of accelerated muscle growth that leads to larger fibers. Klotho also transcriptionally downregulated the H3K27 demethylase Jmjd3, leading to increased H3K27 methylation and decreased expression of genes in the canonical Wnt pathway, which was associated with a delay in muscle differentiation. In addition, Klotho stimulation and Jmjd3 downregulation produced similar but not additive reductions in the expression of Wnt4, Wnt9a, and Wnt10a in myogenic cells, indicating that inhibition occurred through a common pathway. Together, our results identify a novel pathway through which Klotho influences myogenesis by reducing the expression of Jmjd3, leading to reductions in the expression of Wnt genes and inhibition of canonical Wnt signaling.

**Abbreviations**
AEC: 3‐amino‐9‐ethylcarbazole
BAM: binary sequence alignment/map
BP: biological processes
ChIP: chromatin immunoprecipitation
ChIP‐Seq: chromatin immunoprecipitation with sequencing
DAVID: database for annotation, visualization, and integrated discovery
EFmKL46: human elongation factor‐1alpha promoter
Ezh2: enhancer of zeste 2 polycomb repressive complex 2 subunit
GO: gene ontology
H3K27: histone 3 lysine 27
H3K27me2/3: di‐methylated or tri‐methylated histone 3 lysine 27
H3K27me3: tri‐methylated histone 3 lysine 27
Jarid2: Jumonji, AT‐rich interactive domain 2
Jmjd3: Jumonji domain‐containing 3, histone lysine demethylase
KEGG: Kyoto encyclopedia of genes and genomes
KL: Klotho
KL Tg/KL Tg+: Klotho transgene
Macs: model‐based analysis of ChIP‐Seq
Myod1: myogenic differentiation 1
Myog: myogenin
Pax7: paired‐box protein 7
PRC2: polycomb repressive complex 2
SAM: sequence alignment/map
sKL: soluble KL
TSS: transcriptional start site
UTX: ubiquitously‐transcribed X chromosome tetratricopeptide, histone lysine demethylase
Wnt: wingless‐type MMTV integration site family, member
αKL: alpha Klotho

## 1 INTRODUCTION

The life‐long health and function of skeletal muscle can be strongly influenced by a population of muscle stem cells that reside in the muscle. These cells, called satellite cells, experience extensive and complex regulation by numerous factors intrinsic to muscle cells, by factors produced by other cells, and by interactions with the extracellular matrix.^1^ Each of those regulatory influences has direct, immediate effects on satellite cells that determine their state of activation, proliferation, and differentiation during muscle development. However, the responses of satellite cells to those signals also have long‐term influences on muscle mass and regenerative capacity that can affect the vitality of organisms.^1^, ^2^, ^3^

Although establishing and maintaining a sufficient population of satellite cells is necessary for normal muscle homeostasis and health throughout life, the period of early postnatal development may be particularly important in determining the life‐long function of muscle. The first 28 days following birth (P28) in mice is an especially dynamic period influencing satellite cell numbers and fate. During that period, ~80% of rodent satellite cells are actively proliferating^4^, ^5^ but by 6 to 8 weeks of age, fewer than 1% of satellite cells are in the cell cycle^6^, ^7^ and the satellite cell pool number is established.^8^ Measurements of changes in satellite cell numbers during muscle development and maturation indicate that growth of muscle fibers in mice until ~P21 may be influenced by the number of satellite cells present.^9^, ^10^ In addition, the adult numbers of satellite cells and muscle fiber nuclei are largely established in mice by ~P28,^9^ although the numbers can be modified in mature organisms by exercise, injury, or disease.^11^, ^12^ Furthermore, reductions of satellite cell numbers at ~P28 that are caused by limb irradiation produce smaller muscle fibers and fewer myonuclei in mice which persist until the mice are at least 14 months old.^13^, ^14^ Thus, factors that regulate satellite cell number and differentiation early in life may have long‐term influences on muscle mass and function because maintaining a sufficient pool of satellite cells is necessary for successful muscle regeneration throughout life.^15^

Satellite cell numbers increase when quiescent, non‐proliferative satellite cells that express the Pax7 transcription factor become activated to a proliferative population that expresses Pax7 and the transcription factor MyoD. MyoD plays a central role in regulating the early stages of muscle differentiation.^16^, ^17^, ^18^ Those Pax7+/MyoD+ cells can continue to proliferate or they can return to a Pax7+/MyoD‐ quiescent state, or they can withdraw from the cell cycle and express myogenin.^19^ Myogenin, also a transcription factor, regulates the terminal differentiation of myogenic cells and their fusion into mature muscle fibers.^20^, ^21^ Thus, any factor that increases the cycling of Pax7+/MyoD+ myogenic cells or inhibits the transition of proliferative myogenic cells to post‐mitotic cells could expand satellite cell numbers during early postnatal development.

Several observations suggest that the anti‐aging protein Klotho could potentially influence the large, rapid increase of Pax7+ satellite cells in early postnatal myogenesis. First, stimulation of myogenic cells in vitro with recombinant Klotho more than doubles their proliferation during 48‐h period.^12^ Also, Klotho hypomorphic mice show large reductions in the number of Pax7+ myogenic cells at P14.^22^ In addition, elevated expression of Klotho in dystrophic muscle causes large increases in satellite cells that persist into late stages of pathology, showing a positive relationship between Klotho expression levels and satellite cell numbers.^12^ Finally, Klotho expression in healthy skeletal muscle is greatest during early postnatal development (P14) and then rapidly declines^12^ over a time course that resembles the reduction of numbers of proliferative satellite cells in postnatal development.^4^, ^5^, ^6^, ^7^, ^10^

In this investigation, we test the hypothesis that high levels of Klotho expression in early postnatal myogenesis increase satellite cell numbers, in part, through effects on the epigenetic regulation of genes that regulate myogenesis. We explore a potential role for Klotho in affecting epigenetic regulatory mechanisms that control the transition of myogenic cells from a proliferative population to a post‐mitotic, terminally‐differentiated population. For example, changes in the expression of enzymes that affect the methylation of lysine 27 on histone 3 (H3K27) located at the regulatory region of specific genes have large influences on myogenic cell proliferation and differentiation. H3K27 methylation can be increased by the polycomb repressive complex 2 (PRC2) which includes the methyltransferase Ezh2 and the regulatory protein Jarid2, leading to gene repression.^23^, ^24^ Deletion or inhibition of either Ezh2 or Jarid2 in myogenic cells reduces Pax7+ cell numbers and disrupts satellite cell activation and differentiation.^25^ Conversely, UTX (KDM6A) and Jmjd3 (KDM6B) demethylate H3K27 to allow transcriptional activation that is essential for muscle terminal differentiation.^26^, ^27^ UTX is an important, positive regulator of myogenin expression in vitro,^28^ and deletion of *Utx* in satellite cells inhibits the expression of myogenin following muscle injury.^29^ Perturbing Jmjd3 expression also affects the expression of myogenic transcription factors; the transient, ectopic expression of Jmjd3 in pluripotent stem cells induces expression of Pax7.^30^, ^31^ Thus, if Klotho modifies the expression or activity of any of the key epigenetic regulatory enzymes that control myogenesis, the high levels of Klotho expression that occur in early postnatal muscle could play important roles in influencing the numbers and development of satellite cells.

## 2 MATERIALS AND METHODS

### 2.1 Mice

C57 BL/6 (wild‐type/Wt mice) were purchased from The Jackson Laboratory (Jax Labs, Bar Harbor, ME, USA) and transgenic mice overexpressing Klotho (KL Tg+) were generously gifted by Dr. Makoto Kuro‐o. The *klotho* transgene is under the control of the constitutively expressed human elongation factor‐1alpha promotor (EFmKL46). Mice overexpressing Klotho were back‐crossed onto the C57 BL/6 background and were genotyped at weaning to ensure the presence of mutant alleles. Mice were housed in a specific pathogen‐free facility under 12‐h light and dark cycles. Only male mice were used in these studies. Mice were euthanized by inhalation of isoflurane and weighed prior to muscle collection. Individual muscles were collected, weighed, and flash‐frozen for subsequent RNA isolation or histological analysis. Experimental group size ranges from 4 to 5 mice per group.

### 2.2 Muscle fiber cross‐sectional area

Frozen quadriceps muscles were cross‐sectioned at the midbelly and stained for 10 min with hematoxylin followed by three, double‐distilled H_2_O rinses. Fiber cross‐sectional area measurements were taken for no fewer than 500 fibers for each section analyzed. Fibers were sampled from five or more separate locations within the muscle cross‐section and digitally measured using ImageJ.^32^, ^33^ Classification of small and large fibers was determined by setting three standard deviations from the mean cross‐sectional area for the control group and quantifying the percent of fibers that fell within those ranges.^34^, ^35^

### 2.3 RNA isolation and quantitative PCR

Whole muscle tissue was mechanically homogenized (Dupont, Wilmington, DE, USA) in Trizol (Invitrogen, Waltham, MA, USA). RNA was extracted with chloroform and precipitated with isopropanol. RNA was DNase‐treated and purified with RNeasy Mini Kit (Qiagen, Hilden, Germany) according to the manufacturer's protocol. The total RNA was quantified by spectrophotometry (Beckman, Brea, CA, USA) at 260 nm absorbance. RNA samples used for analysis had a concentration greater than or equal to 0.2 µg/µl and an absorbance ratio of 1.8 or higher. RNA quality was determined by the clear separation of 28S and 18S ribosomal RNA by electrophoresis. Two micrograms of total RNA were reverse transcribed with Super Script Reverse Transcriptase II (Invitrogen, Waltham, MA, USA) using Oligo(dT)_12‐18_ Primers (Invitrogen, Waltham, MA, USA) for product extension. cDNA was used to measure the expression for the genes of interest using SYBR Green qPCR Master Mix (Bio‐Rad, Hercules, CA, USA) or iTaq Universal SYBR Green Supermix (Bio‐Rad, Hercules, CA, USA). Real‐time quantitative PCR was performed on an iQ5 thermocycler system with optical system software (Bio‐Rad, Hercules, CA, USA) or on a QuantStudio 5 system (Thermo Fisher, Waltham, MA, USA). To increase scientific rigor and because genes used to normalize qPCR data can vary with age, disease, or treatments,^36^, ^37^, ^38^ we empirically determined that Srp14, Hprt1, and Rnps1 were suitable reference genes based on methods previously described.^39^ The normalization factor for each sample was calculated using the geometric mean of the Ct‐values measured from the reference genes. The highest relative expression value for each gene was set to 1 and all other expression values were scaled accordingly. QPCR primer sequences are listed in Table 1.

**TABLE 1:** Primers sequences used for PCR

Gene	Forward	Reverse
*Axin2*	GACGCACTGACCGACGATTC	CTGCGATGCATCTCTCTCTGG
*Ccnd1*	CGAGGAGCTGCTGCAAATG	GGGTTGGAAATGAACTTCACATC
*Ezh2*	CTGCTGAGCGTATAAAGACAC	CTTAGAGGAGCTGGACGT
*Fzd3*	GGAACGCTGCAGAGAGTATCAC	GGAATCCCAACTATGAGAGCC
*Fzd9*	TGTGTTGGTACCCCTATCTTGC	CTTCTCCAGCTTCTCCGTATTG
*Hprt1*	GCAAACTTTGCTTTCCCTGG	ACTTCGAGAGGTCCTTTTCACC
*Jarid2*	GGTCTGCTCAGGACTTACGG	TTGGGTTTGGTTTCCTTGAC
*Jmjd3*	AGTGAGGAAGCCGTATGCTG	AGCCCCATAGTTCCGTTTGTG
*Klotho*	GTCTCGGGAACCACCAAAAG	CTATGCCACTCGAAACCGTC
*Myod1*	GAGCGCATCTCCACAGACAG	AAATCGCATTGGGGTTTGAG
*Myog*	CCAGTACATTGAGCGCCTAC	ACCGACTCCAGTGCATTGC
*Pax7*	CTCAGTGAGTTCGATTAGCCG	AGACGGTTCCCTTTGTCGC
*Rnps1*	AGGCTCACCAGGAATGTGAC	CTTGGCCATCAATTTGTCCT
*Srp14*	AGAGCGAGCAGTTCCTGAC	CGGYGCTGATCTTCCTTTTC
*Wnt4*	GAGAAGTTTGACGGTGCCAC	GTCCTCATCTGTATGTGGCTTG
*Wnt9a*	GACTTCCACAACAACCTCGTG	AGGAGCCAGACACACCATG
*Wnt10a*	CGAATGAGACTCCACAACAACCG	CGTGGCATTTGCACTTACGC
*Utx*	GGTGCTTTATGTCGATCCCAG	CAGCATTGGACAAAGTGCAGG

### 2.4 Production of Pax7 antibody

Hybridoma cells expressing antibodies to Pax7 were purchased from the Developmental Studies Hybridoma Bank (DSHB, University of Iowa). Cells were cultured in ventilated T‐75 flasks with a complete medium consisting of Iscove's Modified Dulbecco's Medium (Sigma, St. Louis, MO, USA) supplemented with sodium bicarbonate, 1% penicillin‐streptomycin (Gibco, Waltham, MA, USA), and 20% fetal bovine serum (FBS) according to the DSHB culturing protocol. Complete medium was added every other day until day 6 in the culture at which the time serum‐free complete medium was added to cultures to maintain a cell density between 5 × 10^5^ to 1 × 10^6^ cells/ml. After 14 days in culture, cells were split evenly into non‐ventilated flasks and diluted with equal volumes serum‐free complete medium. After 14 days of culturing, Pax7 conditioned medium was collected and sterile filtered prior to antibody purification. Anti‐Pax7 was affinity‐purified from a conditioned medium and eluted with 0.1 M glycine. Antibody concentration was determined by measuring absorbance at 280 nm with a spectrophotometer (Beckman, Brea, CA, USA). Antibody specificity was determined by western blot and immunohistochemistry.

### 2.5 Immunohistochemistry

Quadriceps muscles were dissected and rapidly frozen in isopentane cooled in liquid nitrogen. Frozen, OCT embedded cross‐sections were cut at a thickness of 10 μm. Sections were air‐dried for 30 min and fixed with 4% paraformaldehyde (PFA) or ice‐cold acetone for 10 min and washed for 15 min in phosphate‐buffered saline (PBS). Prior to labeling with antibodies for Pax7, sections were subject to 40 min of antigen retrieval in sodium citrate buffer containing 0.05% Tween‐20 (pH 6.0) and heated in a water bath to 95–100°C. Endogenous peroxidases were quenched for 10 min with 0.3% H_2_O_2_. Sections were treated with blocking buffer from a mouse‐on‐mouse immunohistochemistry kit (M.O.M Kit; Vector Laboratories, Burlingame, CA, USA) supplemented with 0.3 M glycine for 1 h. Sections were incubated with mouse anti‐dystrophin (1:30; RRID:AB_442081), anti‐Pax7 (1:500), or anti‐MyoD (1:50; RRID:AB_395255) primary antibodies in a humidified chamber, overnight at 4°C. Sections were subsequently incubated with the M.O.M. kit biotin‐conjugated anti‐mouse IgG (1:200) for 30 min, followed by 15 min of PBS washes and a 30‐min incubation with M.O.M. kit ABC reagents. Immunolabeling was visualized with the peroxidase substrate 3‐amino‐9‐ethylcarbazole (AEC kit; Vector Laboratories, Burlingame, CA, USA), causing a dark red reaction product. Following the development, sections labeled for dystrophin were stained with hematoxylin as described above. The number of myonuclei per fiber was determined by counting the number of myonuclei stained for hematoxylin within dystrophin‐stained fibers and the total number of fibers within a field of view. The number of immunolabeled cells per 100 fibers was determined by counting the number of immunolabeled cells and the total number of muscle fibers in muscle cross‐sections.

### 2.6 Immunofluorescence

For sections immunolabeled with two or more antibodies, tissue was fixed with 4% PFA for 10 min, subject to 40‐min antigen retrieval and a 1‐h blocking incubation (M.O.M. kit) with 0.3 M glycine. Sections were co‐labeled with anti‐Pax7 (1:500) or anti‐Pax7 (1:50; RRID:AB_2159836) and goat anti‐Klotho (1:10; RRID:AB_2296612), chicken anti‐laminin (1:200; RRID:AB_2134058), rabbit anti‐Jmjd3 (1:200; RRID:AB_10987745), rabbit anti‐H3K27me3 (1:1000; RRID:AB_2616029), or antibodies probing the active, non‐phosphorylated (Ser45) β‐catenin (1:1500; RRID:AB_2650576). Sections were incubated with primary antibodies overnight in a humidified chamber at 4°C. Sections were subsequently washed and incubated for 30 min with horse anti‐mouse Dylight‐594 (1:200; RRID:AB_2336412) and horse anti‐rabbit Dylight‐488 (1:100; RRID:AB_2336403), anti‐chicken IgY H&L Alexa‐488 (1:200; RRID:AB_2827653), or biotinylated anti‐goat secondary (1:200; RRID:AB_2336123) followed by avidin‐Dylight 488 (1:500, RRID:AB_2336405). Sections were mounted with Prolong Gold Antifade Mountant containing DNA stain DAPI (#P36931; Invitrogen, Waltham, MA, USA). For data expressed as a percent of Pax7+ cells beneath the basal lamina, cells were determined to be Pax7 and DAPI positive and then determined to be beneath the anti‐laminin labeled basal lamina or outside the anti‐laminin labeled basal lamina. For data expressed as a percent co‐labeled, cells were determined to be Pax7 and DAPI positive then determined to be Klotho, Jmjd3, H3K27me3 or β‐catenin positive. Data are expressed as the percentage of total Pax7+ satellite cells that are under laminin or as the total Pax7+ satellite cells that also express Jmjd3, H3K27me3, or active β‐catenin (Jmjd3+ Pax7+, H3K27me3+ Pax7+ or β‐catenin+ Pax7+/ total Pax7+).

### 2.7 Cell culture and in vitro treatments

C2C12 myoblasts were seeded on 60 mm culture plates at 100 000 cells per dish or in 6‐well plates at 40,000 cells per well. Myoblasts were maintained in growth medium (Dulbecco's Modified Eagle Medium (DMEM) containing 10% FBS, penicillin and streptomycin) at 37°C and in 5% CO_2_. The culture medium was refreshed every other day unless otherwise stated. Myogenic cells were serum‐starved to induce differentiation and collected at the 1 day, 5 days, or 7 days following differentiation.

### 2.8 Klotho stimulation of myoblasts in vitro

C2C12 myoblasts were seeded and cultured as outlined above. Cultures were stimulated with 10 μg/ml heparin (Sigma, St. Louis, MO, USA) or heparin and 1 μg/ml Klotho (R&D Systems, Minneapolis, MN, USA) in a growth medium at 24‐ and 48‐h post‐plating. Following 48 h of stimulation, cells were collected in Trizol reagent for RNA isolation.

### 2.9 Klotho stimulation with subsequent siRNA knock‐down of Jmjd3

C2C12 myoblasts were seeded in 6‐well dishes, cultured in antibiotic‐free DMEM containing 10% heat‐inactivated FBS at 37°C and 5% CO_2_. After 24 h in culture, cells were rinsed three times with 1 ml of DPBS and cultured in Opti‐MEM (Gibco, Waltham, MA, USA). Cells were transfected with 50 pmol/ml of Stealth siRNA oligos targeting Kdm6b/Jmjd3 (#1320001; Invitrogen, Waltham, MA, USA) or Stealth siRNA medium GC control oligos (#12935300; Invitrogen, Waltham, MA, USA) using RNAiMAX reagent (Invitrogen, Waltham, MA, USA) for 6 h according to the manufacturer's protocol. Following transfection, cells were collected for RNA or protein analysis.

### 2.10 Jmjd3 overexpression in myogenic cells

C2C12 myoblasts were cultured in 6‐well dishes, as described above. After 24 h in culture, the cells were rinsed three times with 1 ml of DPBS and cultured in Opti‐MEM. Cells were transfected with pCS2‐Jmjd3‐F expression plasmid (RRID:Addgene_17440) or a pCS2 control vector using Lipofectamine 3000 Transfection Reagent (Invitrogen, Waltham, MA, USA) for 6 h according to the manufacturer's protocol. Following transfection, myoblasts were cultured in a differentiation medium for 72‐h and RNA and protein were collected for analysis.

### 2.11 GSK‐J4 treatment of C2C12 myoblasts

C2C12 myoblasts were seeded on 6‐well plates and treated with 1.2 mM of GSK‐J4^40^, ^41^ (Cayman Chemical, Ann Arbor, MI, USA) at 24‐ and 48‐h post‐plating. Following 48 h of treatment, cells were collected for RNA analysis.

### 2.12 Western blot following differentiation

C2C12 myoblasts were cultured to specified confluency and subject to differentiation. Cells were washed three times with ice‐cold DPBS and collected in reducing sample buffer (80 mM Tris‐HCl, pH 6.8, 0.1 M DTT, 70 mM SDS and 10% glycerol) supplemented with proteinase inhibitor cocktail (#P8340; Sigma, St. Louis, MO, USA), 0.2 M Na_3_VO_4_, and 5 M NaF and passed through a 23‐gauge needle five or more times. Cell lysates were boiled for 3 min and centrifuged at 12 000 g for 1 min at 4°C. A portion of the supernatant fraction was used to determine total protein concentration by filter paper assay. Protein homogenates containing 30 μg of total protein were separated on a 10% SDS‐PAGE gel and transferred by electrophoresis to a nitrocellulose membrane for 3 h in transfer buffer (0.2 M glycine, 25 mM Tris base, and 20% methanol). Equal loading and efficiency of transfer were verified by staining with Ponceau S solution (#P‐7170; Sigma, St. Louis, MO, USA). Nitrocellulose membranes were incubated in blocking buffer containing 0.1% Tween‐20, 0.2% gelatin, and 3% dry milk overnight at 4°C. Membranes were probed with anti‐Jmjd3 (1:100), anti‐Klotho (1:50), or anti‐myogenin (1:100) for 3 h at room temperature or overnight at 4°C, washed six times for 10 min in wash buffer (0.05% Tween‐20, 0.2% gelatin, and 3% dry milk) or in wash buffer containing 25 mM Tris, pH 7.4, 0.15 M NaCl (TBS) containing 0.05% Tween‐20 (0.05% TBST) and overlayed with ECL horseradish peroxidase anti‐rabbit IgG (1:100 000; RRID:AB_772206) or ECL horseradish peroxidase anti‐mouse IgG (1:10,000; RRID:AB_772210) for 1 h at room temperature. Membranes were washed six times for 10 min in wash buffer prior to development. Membranes were developed with FemtoGlow Western Plus (#FWPD02; Michigan Diagnostics, Royal Oak, MI, USA) and imaged on a SynGene PXi imager (Bangalore, Karnataka, India) using GeneSys V1.5.4.0 software. Relative quantities of Jmjd3 and myogenin proteins were determined using ImageJ software and normalized to input protein.

### 2.13 Western blot following Jmjd3 inhibition with Klotho and siRNA

After 48 h of Klotho treatment followed by a 6‐h transfection with siRNA targeting Jmjd3, C2C12 cells were washed three times with ice‐cold DPBS and collected in reducing sample buffer supplemented with proteinase inhibitor cocktail, 0.2 M Na_3_VO_4_, and 5 M NaF and passed through a 23‐gauge needle five or more times. Cell lysates were then prepared and analyzed by western blotting as described above, using anti‐Jmjd3 (1:100) or rabbit anti‐desmin (1:50; RRID:AB_476910). Primary antibodies were applied to the blots for 3 h at room temperature. Prior to incubation with Wnt‐related antibodies probing for rat anti‐Wnt4 (1:200; RRID:AB_2215448), rabbit anti‐Wnt9a (1:500; RRID:AB_2772907), or rabbit anti‐Wnt10a (1:500; RRID:AB_1277809), membranes were incubated overnight at 4°C in blocking buffer containing 25 mM Tris pH 7.4, 0.15 M NaCl, 0.1% Tween 20 and 3%–5% dry milk. The following day, membranes were washed in 0.1% TBST wash buffer three to six times for 10 min and probed with primary antibodies in a humidified chamber overnight at 4°C. Following primary incubation, membranes were washed in 0.1% TBST three to six times. Membranes probed with anti‐Wnt4 were overlaid with ECL horseradish peroxidase anti‐rat IgG (1:10 000; RRID:AB_772207) for 1 h at room temperature. Membranes probed with anti‐Wnt9a or anti‐Wnt10a were overlaid with ECL horseradish peroxidase anti‐rabbit IgG for 1 h at room temperature. All membranes were washed three to six times in 0.1% TBST, developed with FemtoGlow Western Plus, and imaged on a SynGene PXi imager (Bangalore, Karnataka, India).

### 2.14 Chromatin immunoprecipitation on Klotho treated myoblasts

C2C12 myoblasts were seeded at 2.0 × 10^5^ on 100‐mm culture dishes maintained in growth medium and treated with Klotho as outlined above. Following 48 h of stimulation, cells were washed with DPBS, released with 0.05% trypsin EDTA (Gibco, Waltham, MA, USA), and quenched with a growth medium. Cells were fixed in 1% formaldehyde on an end‐to‐end rotator (Barnstead/Thermolyne) for 10 min at room temperature. 1% formaldehyde solution was quenched with 2 M glycine for a final concentration of 125 mM glycine and incubated on rotation for 10 min. Cells were washed 3 times with cold DPBS prior to lysing. Subsequent steps were done following the ChIP‐IT High Sensitivity Kit (Active Motif, Carlsbad, CA, USA) manufacturer's protocol. Cells were lysed in chromatin prep buffer (Active Motif, Carlsbad, CA, USA) containing proteinase inhibitor cocktail and 100 mM phenylmethylsulfonyl fluoride (PMSF) and incubated on ice for 10 min. The lysate was transferred to an ice‐cold Dounce homogenizer for mechanical dissociation. The homogenate was then centrifuged at 2350 rpm for 3 min at 4°C. The pellet fraction was resuspended in ChIP buffer containing proteinase inhibitor cocktail and PMSF, transferred to a 1.5 ml sonication tube (Active Motif, Carlsbad, CA, USA), and incubated on ice for 10 min. Chromatin was sheared by sonication (Active Motif, Carlsbad, CA, USA) at 20 amp for cycles of 15 s on and 15 s off to reach a fragmented side of approximately 200 bp. DNA fragments were electrophoresed on a 2.0% agarose gel and digitally visualized (SynGene, Bangalore, Karnataka, India) with gel red staining. ~17 μg of chromatin were incubated with ChIP‐verified anti‐H3K27me2/3 (RRID:AB_2793246) or IgG negative control antibodies on end‐to‐end rotation overnight at 4°C. The following day, Protein G agarose beads were washed and added to each sample for chromatin immunoprecipitation (ChIP). The chromatin‐bead mixtures were incubated for 3.5 h on an end‐to‐end rotator at 4°C. Each sample was loaded onto a ChIP filtration column, washed, and dried by centrifugation at 1250 g for 3 min at room temperature. ChIP DNA was eluted twice with 50 μl of Elution Buffer AM4. ChIP‐DNA was reverse cross‐linked and purified with the ChIP‐IT DNA Isolation Kit (Active Motif, Carlsbad, CA, USA) per the manufacturer's protocol. Briefly, eluted ChIP DNA was mixed with Proteinase K and incubated in a thermomixer (Eppendorf, Hamburg, Germany) set to at 900 rpm and 55°C for 30 min, followed by 80°C for 2 h. DNA was diluted with DNA Purification Binding Buffer with 10 μl of 3 M sodium acetate for pH adjustment yielding a bright yellow reaction mixture. Each sample was placed in a DNA purification column and washed with DNA purification wash buffer. Purified DNA was eluted in 40 μl of DNA purification elution buffer and stored at −20°C prior to DNA sequencing.

### 2.15 Chromatin immunoprecipitation‐sequencing (ChIP‐Seq) analysis

DNA quality control and sequencing were done at the UCLA Technology Center for Genomics and Bioinformatics at the University of California, Los Angeles. Single‐end DNA sequencing was performed on an Illumina (San Diego, CA, USA) HiSeq3000 instrument with ~39 to 45 million reads per sample and a read length of 50 base pairs (bp). Raw fastq files were aligned to the mm10 genome using Bowtie2^42^ with default parameters, achieving an alignment rate between 95 and 98%. The resulting SAM files were converted to BAM format and sorted using Samtools.^43^ Broad peaks were called using Model‐based analysis of ChIP‐Seq^44^ (macs2 callpeak function) with the sorted ChIP and input alignments (BAM files) as the treatment and control files, respectively, and specifying the following parameters: ‐‐broad ‐‐broad‐cutoff 0.1 ‐g mm ‐‐nomodel. We calculated peaks found after Klotho treatment but not in a control condition, and quantified read density around peak centers (±1 kb) using computeMatrix from DeepTools^45^ with the ‐‐skipZeros parameter and the output of bamCoverage^45^ as inputs. The resulting matrix was then used with plotHeatmap^45^ for visualization. Genomic regions and functional analyses were done using R on peaks falling within −3000 to +300 bp from the transcription start site (TSS) of genes defined by the bioconductor^46^, ^47^ package TxDb.Mmusculus.UCSC.mm10.ensGene.^48^ We used R to identify gene promoters that overlap with H3K27me2/3 peaks and quantify the percent overlap. Promoters with H3K27me2/3 peak occupancy after Klotho treatment only are shown in Table 2 and by definition have 0% H3K27me2/3 overlap in the vehicle‐treated control condition. Gene ontology (GO) analysis and Kyoto encyclopedia of genes and genomes (KEGG)^49^ was done using the database for annotation, visualization, and integrated discovery (DAVID)^50^, ^51^ which uses a modified Fisher's Exact test to examine the statistical significance of enrichment for each term. KEGG results were verified using the KEGG.db package from Bioconductor and a hypergeometric test to measure statistical significance for each term. Raw ChIP‐seq data were uploaded to the National Center for Biotechnology Information's Gene Expression Omnibus and are available under accession number GSE189109.

**TABLE 2:** Wnt‐related genes whose promoters (3000 bp upstream to 300 bp downstream of TSS) overlap with H3K27me2/3 peaks in Klotho‐stimulated C2C12 myoblasts

Symbol	Chr:Start‐End	Gene ID	H3K272/3 peak(s) in KL‐treated cells	Promotor overlap (%)
*Wnt4*	chr4:137277489‐137299726	ENSMUSG00000036856	chr4:137274634‐137274877; chr4:137276224‐137277069	33
*Wnt9a*	chr11:59306928‐59333552	ENSMUSG00000000126	chr11:59304349‐59305664	40
*Wnt10a*	chr1:74791516‐74804179	ENSMUSG00000026167	chr1:74789946‐74790493	17
*Fzd3*	chr14:65201026‐65262463	ENSMUSG00000007989	chr14:65261701‐65263591	43
*Fzd9*	chr5:135248938‐135251230	ENSMUSG00000049551	chr5:135252831‐135253153	10
*Frzb*	chr2:80411970‐80447625	ENSMUSG00000027004	chr2:80446165‐80447428	3
*Wisp3*	chr10:39150971‐39163794	ENSMUSG00000062074	chr10:39164763‐39165022; chr10:39166089‐39167811	29
*Csnk2a2*	chr8:95446096‐95488820	ENSMUSG00000046707	chr8:95491364‐95492299	14
*Pp2cb*	chr8:33599621‐33619794	ENSMUSG00000009630	chr8:33597564‐33598858	39
*Sfrp2*	chr3:83766321‐83774316	ENSMUSG00000027996	chr3:83766030‐83766774	18
*Csnk1e*	chr15:79417856‐79443919	ENSMUSG00000022433	chr15:79444679‐79446189	46
*Nkd2*	chr13:73818534‐73847631	ENSMUSG00000021567	chr13:73848780‐73850045	38
*Nkd1*	chr8:88521344‐88594887	ENSMUSG00000031661	chr8:88520969‐88524273	20
*Shisa2*	chr14:59625281‐59631658	ENSMUSG00000044461	chr14:59625308‐59626307	8

Note

Promoter overlap percentage indicates the percent of a given promoter overlapping with H3K27me2/3 peak(s) in the Klotho‐treated condition. Notably, all listed promoters have 0% overlap with H3K27me2/3 peaks in the vehicle‐treated control condition, which suggests that these Wnt‐related promoters have preferential heterochromatic mark deposition under Klotho stimulation. Column names indicate: gene symbol, gene location (chr:start‐end), Ensemble gene ID, location of broad peaks, and promotor overlap (%) with H3K27me2/3 peaks in C2C12 cells treated with recombinant Klotho for 48 h.

### 2.16 Statistics

Data are presented as the mean ± standard error of the mean (SEM). Non‐parametric student's *t*‐test was used when determining differences between two groups and one‐way analysis of variance (ANOVA) with Dunnett's multiple comparison test when comparing more than one group to one control group or Tukey's post hoc was used when comparing more than two groups. Groups were determined to be significantly different at *p* < .05.

## 3 RESULTS

### 3.1 Klotho modulates muscle development during early postnatal growth

**FIGURE 1:**
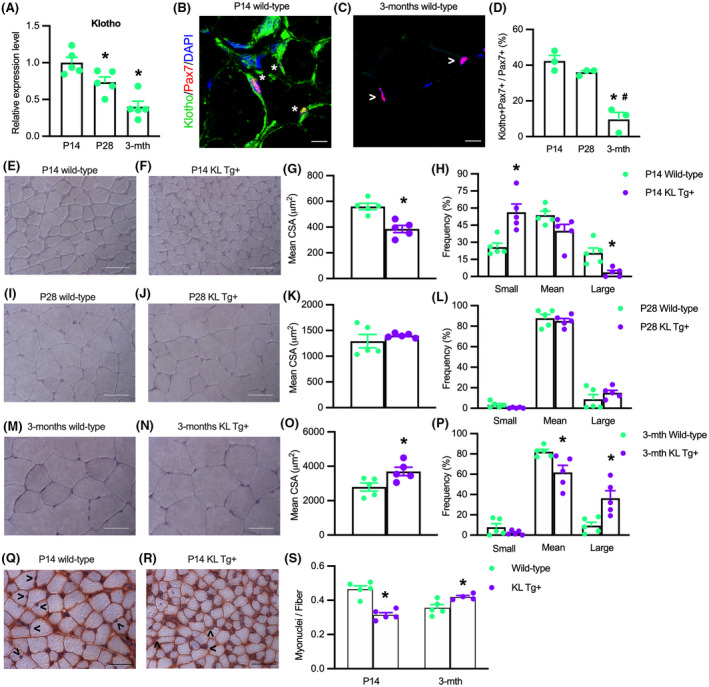
Expression of a KL Tg affects muscle development. (A) QPCR data showing relative mRNA expression of *klotho* in quadriceps muscle lysates of P14, P28, and 3 months Wt mice. *N* = 5 per time point. *Indicates significantly different from P14 at *p* < .05 analyzed by one‐way ANOVA followed by Dunnett's multiple comparisons test. Error bar represents SEM. (B, C) Sections of P14 and 3 months Wt quadriceps muscles labeled with anti‐Pax7 (red), anti‐Klotho (green), and DNA labeled with DAPI (blue). *Indicates Pax7+ cells that are also Klotho+. Open arrowhead (>) indicates Pax7+ single‐labeled cells. Bars = 10 μm. (D) Ratio of Klotho+/Pax7+ cells to total Pax7+ cells in sections of quadriceps muscles. (E,F) Representative images of Wt (E) and KL Tg+ (F) quadriceps muscle at P14 stained with hematoxylin. Bar = 50 μm. (G) Mean cross‐sectional area for quadriceps muscle fibers from P14 Wt and KL Tg+ mice. (H) Frequency distribution of fiber cross‐sectional areas for quadriceps muscles at P14 from Wt and KL Tg+ mice. *N* = 5. (I, J) Representative images of Wt (I) and KL Tg+ (J) quadriceps muscle at P28 stained with hematoxylin. Bar = 50 μm. (K) Mean cross‐sectional area for quadriceps muscle fibers from P28 Wt and KL Tg+ mice. (L) Frequency distribution of fiber cross‐sectional areas for quadriceps muscles at P28 from Wt and KL Tg+ mice. (M, N) Representative images of Wt (M) and KL Tg+ (N) quadriceps at 3 months stained with hematoxylin. Bar = 50 μm. (O) Mean cross‐sectional area for quadriceps muscle from 3 months Wt and KL Tg+ mice. (P) Frequency distribution of fiber cross‐sectional areas for quadriceps muscles at 3 months from Wt and KL Tg+ mice. For G, K, and O, * indicates significantly different from the mean cross‐sectional area of age‐matched Wt fibers at *p* < .05 analyzed by *t*‐test. Error bar represents SEM. *N* = 5. For H, L, and P, * indicates significantly different from age‐matched Wt fibers of the same sized group at *p* < .05 analyzed by *t*‐test. Error bar represents SEM. *N* = 5. (Q, R) Representative images of Wt (Q) KL Tg+ (R) quadriceps at P14 stained with anti‐dystrophin and hematoxylin. Open arrowhead (>) indicates myonuclei. Bar = 50 μm. (S) Numbers of myonuclei per fiber in quadriceps from Wt and KL Tg+ mice at P14 and 3 months. *Indicates significantly different from age‐matched Wt fibers at *p* < .05 analyzed by *t*‐test. Error bar represents SEM. *N* = 5

Klotho expression in skeletal muscle declines from early postnatal development until maturity, which suggests that Klotho may affect muscle development in young animals.^12^ Our findings show that *klotho* mRNA expression is highest in wild‐type mice at 14 days after birth (P14), declines during the mid‐stage of postnatal development at 28 days after birth (P28) and is further reduced in 3 months old adult muscles (Figure 1A). Immunohistological observations at P14 show that Klotho protein is located in Pax7‐expressing cells (Pax7+), on the surface of some myofibers, and in other cells in the interstitium (Figure 1B). However, by 3 months of age, the proportion of Pax7+ cells that expressed Klotho significantly declined (Figure 1C,D), confirming that the reductions of Klotho mRNA levels in the muscle that occurred between P14 and 3 months of age (Figure 1A) were mirrored by reductions in the proportion of Pax7+ cells that expressed detectible levels of Klotho protein. Because Klotho protein is expressed in Pax7+ cells and muscle fibers during development and *klotho* expression declines during postnatal muscle growth, we tested whether modifying *klotho* expression would affect muscle growth in young mice (Figure S1). QPCR data confirmed that the *klotho* transgene (KL Tg) produced elevated levels of *klotho* mRNA during skeletal muscle development and in adult skeletal muscle (Figure S1A). However, the expression of the transgene had only slight effects on reducing total body mass or the mass of individual muscles at P14 and no effect on body or muscle mass at P28 or 3 months old (Figure S1B–H).

Despite the small effects of KL Tg expression on muscle mass in young mice, we observed significant effects on muscle fiber growth. The mean cross‐sectional area of quadriceps muscle fibers was reduced by more than 30% in KL Tg+ mice during early postnatal development (Figure 1E–H). However, fiber size did not differ between KL Tg+ and wild‐type mice at P28 (Figure 1I–L) and the fiber size of KL Tg+ mice exceeded wild‐type fibers by more than 24% at 3 months of age (Figure 1M–P). These changes in fiber cross‐sectional areas represented a ~4‐fold increase in wild‐type muscles and a ~10‐fold increase in KL Tg+ fibers between P14 and 3 months of age (Figure 1G,O). Collectively, these data show that increased expression of *klotho* during early postnatal growth delays muscle development, but subsequently the transgene accelerates muscle growth.

We also tested whether the differences in muscle fiber cross‐sectional area between transgenic and control mice were reflected by differences in myonuclei per fiber by assaying whether transgene expression affected the number of hematoxylin‐stained myonuclei in anti‐dystrophin‐stained sections. Our measurements show that at P14 when transgenic muscle fibers have smaller CSA (Figure 1G), there are fewer myonuclei per fiber (Figure 1Q–S). At 3 months, when transgenic muscle fibers have greater CSA (Figure 1O), there are more myonuclei per fiber (Figure 1S).

### 3.2 Klotho increases numbers of activated satellite cells during postnatal development

**FIGURE 2:**
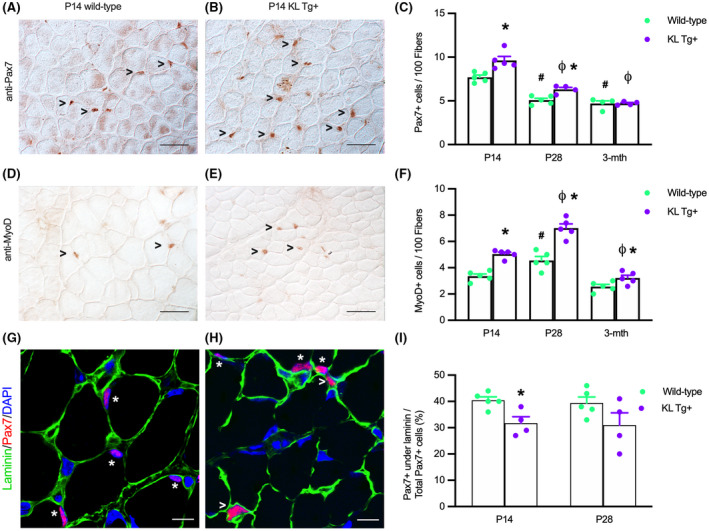
KL Tg expression increases numbers of satellite cells and activated myoblasts during early postnatal development (A, B) Representative images of Wt (A) and KL Tg+ (B) quadriceps muscle at P14 immunolabeled for Pax7 (reddish‐brown nuclei). (C) Numbers of Pax7+ cells per 100 fibers in quadriceps from Wt and KL Tg+ at P14, P28, and 3 months mice. (D, E) Representative images of Wt (D) and KL Tg+ (E) quadriceps muscle at P14 immunolabeled for MyoD (reddish‐brown nuclei). (F) Numbers of MyoD+ cells per 100 fibers in quadriceps from Wt and KL Tg+ at P14, P28, and 3 months mice. For A, B, D, and E, open arrowheads (>) indicate Pax7+ (A, B) or MyoD+ (D, E) labeled cells. Bar = 50 μm. For C and F, *indicates significantly different from age‐matched Wt control at *p* < .05 analyzed by *t*‐test. ^#^Indicates significantly different from P14 Wt at *p* < .05 analyzed by one‐way ANOVA with Tukey's multiple comparisons test. ^ϕ^Indicates significantly different from P14 KL Tg+ at *p* < .05 analyzed by one‐way ANOVA with Tukey's multiple comparisons test. Error bar represents SEM. *N* = 4 or 5. (G, H) Representative images of Wt (G) and KL Tg+ (H) quadriceps muscle at P14 immunolabeled for Pax7 (red), laminin (green), and DNA labeled with DAPI (blue). *Indicates Pax7+ cells under the basal lamina. Open arrowheads (>) indicate Pax7+ cells outside of laminin. Bars = 10 μm. (I) Ratio of Pax7+ cells under the basal lamina to total Pax7+ cells in sections of quadriceps muscles from P14 and P28 Wt and KL Tg+ mice. *Indicates significantly different from age‐matched Wt control at *p* < .05 analyzed by *t*‐test. Error bar represents SEM. *N* = 4 or 5

Because the number of satellite cells that are present in muscle in the first weeks of postnatal muscle development can influence the growth of muscle fibers,^9^, ^10^ we tested whether KL Tg expression affected numbers of quiescent or activated satellite cells that expressed Pax7. We found that elevated levels of KL Tg expression increased the number of Pax7+ cells at P14 and P28, but not at 3 months of age (Figure 2A–C). Notably, the reduction of Pax7+ cells in wild‐type muscles that occurred between P14 and 3 months coincided with the reduction of *klotho* expression that occurred between P14 and 3 months (Figure 1A). Similarly, the presence of the transgene increased the number of activated myoblasts indicated by elevated numbers of MyoD+ cells at P14, P28, and at 3 months (Figure 2D–F). We also tested whether KL Tg expression affected the proportion of Pax7+ cells that are located underneath the basal lamina, using double‐immunohistochemistry for Pax7 and laminin (Figure 2G,H,I). These data show that at P14 the proportion of Pax7+ cells that are beneath the basal lamina is reduced by the KL Tg, although transgene expression at that age increased the numbers of Pax7+ cells (Figure 2C), supporting our interpretation that expression of the transgene in young neonatal animals increases the numbers of activated, Pax7+ cells.

### 3.3 Klotho reduces the expressionof the H3K27 demethylase *Jmjd3* in myogenic cells in vitro

**FIGURE 3:**
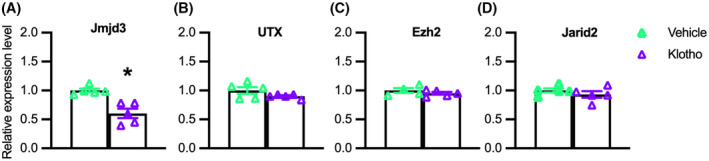
*Klotho* reduces *Jmjd3* expression in myogenic cells in vitro. QPCR data showing relative expression of *Jmjd3* (A), *Utx* (B), *Ezh2* (C), and *Jarid2* (D) in cultured myoblasts treated with recombinant Klotho for 48‐h. *Indicates significantly different from vehicle‐treated cells at *p* < .05 analyzed by *t*‐test. Error bar represents SEM. *N* = 4 or 5 for each data set

We tested whether the effects of Klotho on early myogenesis could result from influences on the expression of epigenetic regulatory factors that may contribute to silencing or activating myogenic genes. In particular, we assayed whether Klotho stimulation of myoblasts in vitro affected the expression of proteins that control the methylation of H3K27 because H3K27 methylation is a well‐established, negative regulator for the expression of myogenic transcription factors that include *Pax7*, *Myod1*, and *Myog*.^28^, ^52^, ^53^ QPCR analysis showed that Klotho stimulation significantly reduced the expression of *Jmjd3*, an H3K27 demethylase (Figure 3A). However, the expression of *Utx* (another H3K27 demethylase), *Jarid2* (which promotes the methylation of H3K27), and *Ezh2* (an H3K27 methyltransferase) were not affected at the mRNA level by Klotho stimulation (Figure 3B–D).

### 3.4 Jmjd3 promotes muscle differentiation in vitro

**FIGURE 4:**
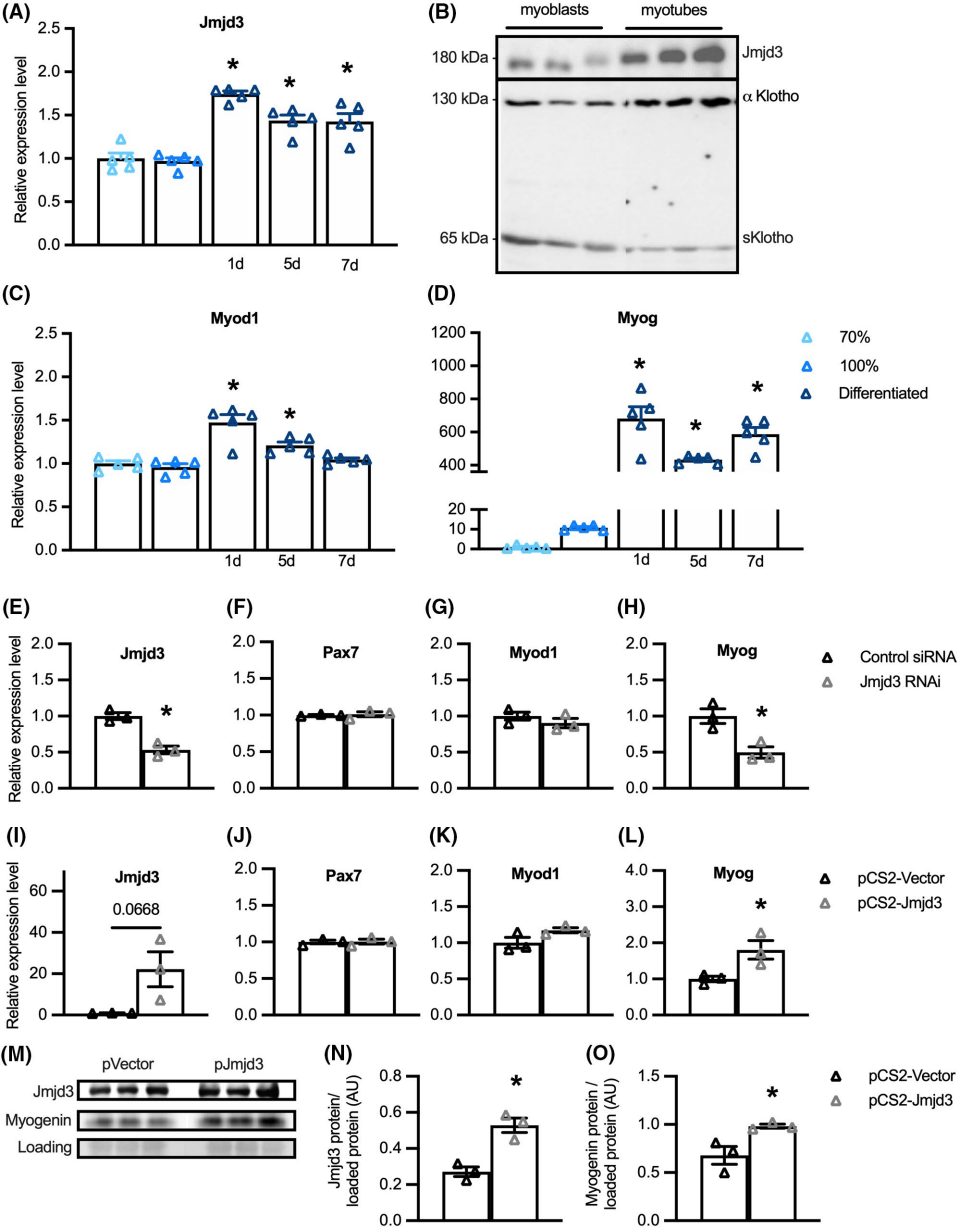
Jmjd3 promotes muscle differentiation in vitro. (A) QPCR data showing relative expression of *Jmjd3* in sub‐confluent (70%) and confluent (100%) myoblasts and in myogenic cells at 1 day, 5 days, and 7 days following the onset of differentiation. *Indicates significantly different from 70% confluent myoblast control group at *p* < .05 analyzed by one‐way ANOVA with Dunnett's multiple comparison test. Error bar represents SEM. *N* = 5 for each data set. (B) Western blot showing relative expression of Jmjd3 and KL in sub‐confluent myoblasts and differentiated myotubes. (C, D) QPCR data showing relative expression of *Myod1* (C) and *Myog* (D) in myogenic cell cultures. *Indicates significantly different from 70% confluent myoblast control group at *p* < .05 analyzed by one‐way ANOVA with Dunnett's multiple comparison test. Error bar represents SEM. *N* = 5 for each data set. (E–H) QPCR data showing relative expression for *Jmjd3* (E), *Pax7* (F), *Myod1* (G), and *Myog* (H) in cultured myoblast cells transfected with control siRNA or siRNA targeting Jmjd3. *Indicates significantly different from cells transfected with control siRNA at *p* < .05 analyzed by *t*‐test. Error bar represents SEM. *N* = 3 for all data sets. (I–L) QPCR data showing relative expression for transcripts of *Jmjd3* (I), *Pax7* (J), *Myod1* (K), and *Myog* (L) in cultured myoblasts transfected with control pCS2‐vector plasmid or pCS2‐Jmjd3‐F expression plasmid for 6‐h followed by 72‐h in differentiation conditions. *Indicates significantly different from cells transfected with control pCS2‐vector plasmid at *p* < .05 analyzed by *t*‐test. Error bar represents SEM. *N* = 3 for all data sets. (M) Western blot showing relative levels of Jmjd3 and myogenin in myogenic cells transfected with control pCS2‐vector plasmid or pCS2‐Jmjd3‐F expression plasmid for 6‐h followed by 72‐h in differentiation conditions. (N, O) Quantification of total Jmjd3 protein (N) or myogenin protein (O) relative to protein loaded per gel lane. *Indicates significantly different from cells transfected with control pCS2‐vector plasmid at *p* < .05 analyzed by *t*‐test. Error bar represents SEM. *N* = 3 for all data sets

Our findings showing that Klotho affects muscle development and decreases *Jmjd3* expression suggested the possibility that some of Klotho's effects on myogenesis could be mediated by its downregulation of Jmjd3. Several observations support that possibility. First, qPCR data show that *Jmjd3* expression increased at the onset of muscle differentiation and then remained elevated for at least 7 days (Figure 4A). In addition, western blots showed more Jmjd3 protein in myotubes than in myoblasts (Figure 4B) and the increase in Jmjd3 in myotubes coincided with a shift in the expression of Klotho isoforms. Western blot probing for full‐length, transmembrane αKlotho (αKL) and truncated, soluble Klotho (sKL) showed sKL is the dominant form of Klotho in myoblasts and αKL is highly upregulated in myotubes (Figure 4B). We also observed that the downregulation of sKL and upregulation of Jmjd3 during myogenesis coincided with a small increase in *Myod1* expression (Figure 4C) and a greater than 680‐fold increase in *Myog* expression (Figure 4D), linking elevations of Jmjd3 levels in myogenesis with increased terminal differentiation of myogenic cells.

We then assayed whether Jmjd3 may have influenced these changes in expression of myogenic transcription factors by treating myoblasts with siRNA that targeted the gene sequence encoding the catalytic domain of the Jmjd3 demethylase protein. *Jmjd3* down‐regulation in siRNA treated myoblasts was confirmed by qPCR (Figure 4E). Although *Pax7* mRNA (Figure 4F) and *Myod1* mRNA (Figure 4G) levels were unaffected by Jmjd3 inhibition, *Myog* transcripts were reduced by more than 50% (Figure 4H). We also assayed whether increased expression of Jmjd3 affected the expression of myogenic transcription factors by transfecting myoblasts with a pCS2‐Jmjd3‐F expression plasmid or a pCS2‐control plasmid. Overexpression of *Jmjd3* (Figure 4I) produced no change in the expression of *Pax7* (Figure 4J) or *Myod1* mRNAs (Figure 4K) but increased *Myog* by nearly twofold (Figure 4L). Similarly, transfection of myoblasts with the Jmjd3 expression plasmid increased Jmjd3 protein relative to total protein compared to myoblasts transfected with control plasmid and likewise increased myogenin protein in Jmjd3 overexpressing myoblasts (Figure 4M–O). Together, these observations indicate that Jmjd3 positively modulates myogenesis as muscle cells transition from proliferative, MyoD+ myoblasts into differentiated, myogenin‐expressing cells.

### 3.5 Klotho treatment of myoblasts promotes H3K27 methylation and reduces expression of Wnt‐family genes

**FIGURE 5:**
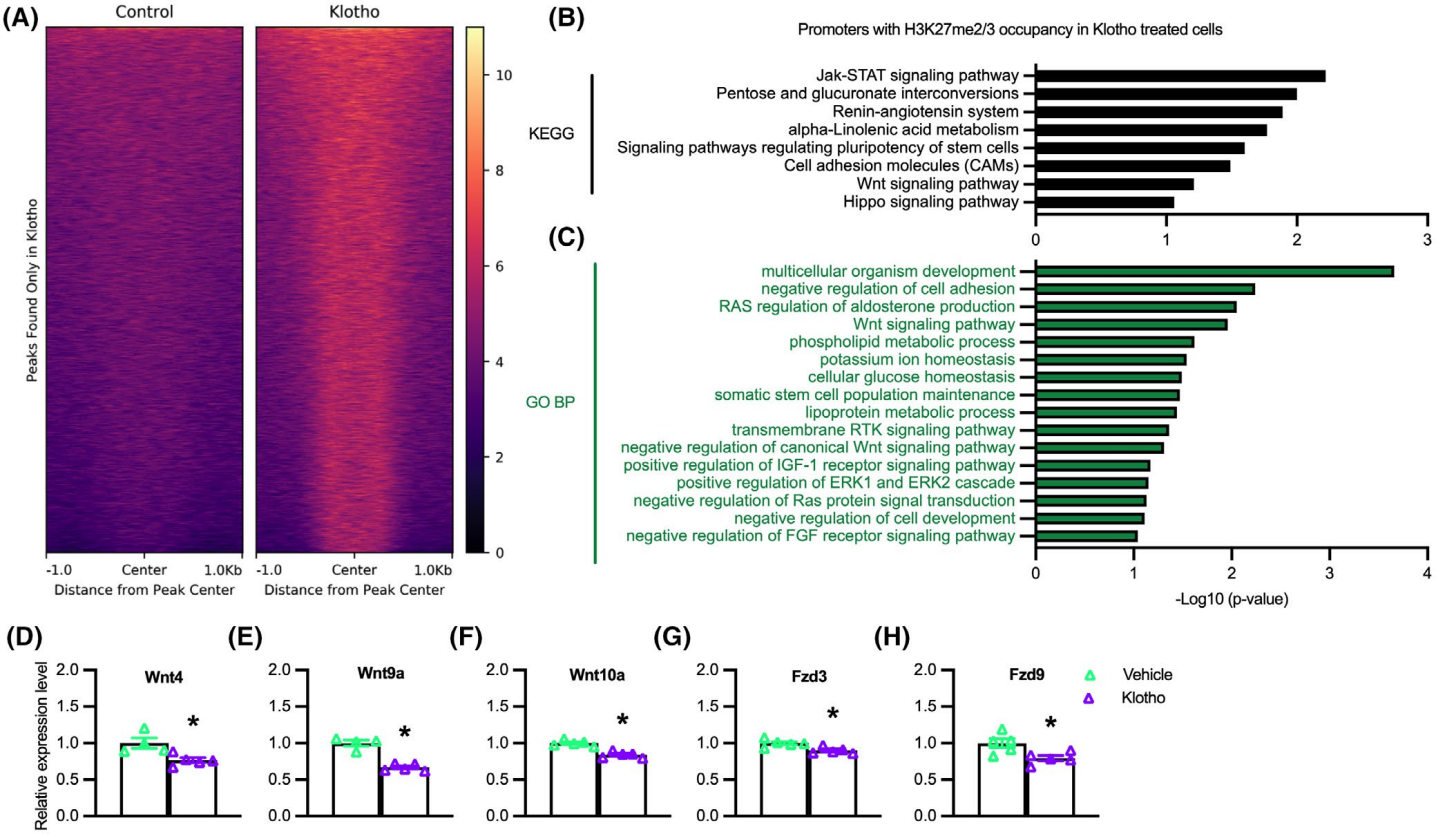
Klotho treatment of myoblasts increases H3K27 methylation and reduces expression of Wnt‐family genes. (A) H3K27me2/3 ChIP‐seq heatmap centered around peaks observed after Klotho treatment that are not observed in the Control, showing higher read density in the Klotho condition. Color scale indicates low (black) to high (pale yellow) read density. (B) KEGG analysis of genes with promoter H3K27me2/3 occupancy in the presence of recombinant Klotho. −log_10_(*p*‐value) indicates the significance of the hypergeometric test results based on the number of gene promoters identified in each category relative to the total number of genes within each term. *N* = 1 for each ChIP and input sample. (C) GO Biological Process analysis of genes with promoter H3K27me2/3 occupancy in the presence of recombinant Klotho treatment. *N* = 1 for each ChIP and input sample. Data graphed as −log_10_(*p*‐value) based on the number of gene promoters identified in each category relative to the total number of genes within each term. (D–H) QPCR data showing relative expression of *Wnt4* (D), *Wnt9a* (E), *Wnt10a* (F), *Fzd3* (G), and *Fzd9* (H) in cultured myoblasts treated with recombinant Klotho for 48‐h. *Indicates significantly different from vehicle‐treated cells at *p* < .05 analyzed by *t*‐test. Error bar represents SEM. *N* = 4 or 5 for each data set

Our observations showing that Klotho is a negative regulator of *Jmjd3* expression and that Jmjd3 is a positive regulator of *Myog* expression suggested that Klotho could possibly affect myogenesis by influencing the H3K27 methylation at myogenic regulatory genes, especially myogenin. We tested whether Klotho influences the methylation state of H3K27 in myoblasts using chromatin immunoprecipitation followed by DNA sequencing (ChIP‐seq) but found no evidence of changes in H3K27 di‐ and tri‐methylation of nucleosomes occupying *Myog*. However, a heat map showing H3K27me2/3 ChIP‐seq peaks that appear after Klotho treatment but not in control samples (Figure 5A), demonstrated significant enrichment of the H3K27me2/3 signal around multiple, other loci. Prominent among those loci, Klotho‐stimulated myoblasts had more H3K27me2/3 silencing marks at the promoter regions of genes classified by KEGG as part of the renin‐angiotensin system, Jak‐STAT signaling, sugar and lipid metabolism, pluripotent stem cell regulation, the Hippo signaling pathway, and the Wnt signaling pathway (Figure 5B). Gene Ontology (GO) analysis for the biological process (BP) terms of genes with increased H3K27 methylation following Klotho stimulation were associated with regulation of developmental processes, stem cell regulatory processes, ion, and metabolic homeostasis, the Ras signaling pathway, and the canonical Wnt signaling pathway (Figure 5C). The accumulation of H3K27me2/3 silencing marks at the promoter regions of Wnt family genes in Klotho‐treated samples could be developmentally important because silencing those genes could disrupt normal myogenesis.^54^, ^55^, ^56^, ^57^ QPCR analysis showed significant reductions in expression of 3 Wnt ligands (*Wnt4*, *Wnt9a*, *Wnt10a*) and 2 Wnt receptors (*Fzd3* and *Fzd9*) in cells stimulated with Klotho (Figure 5D–H), confirming that increased H3K27 methylation at these Wnt‐family genes is associated with their suppression in muscle cells.

### 3.6 Inhibition of H3K27 demethylases reduces expression of Wnt4 and Wnt10a in myogenic cells

**FIGURE 6:**
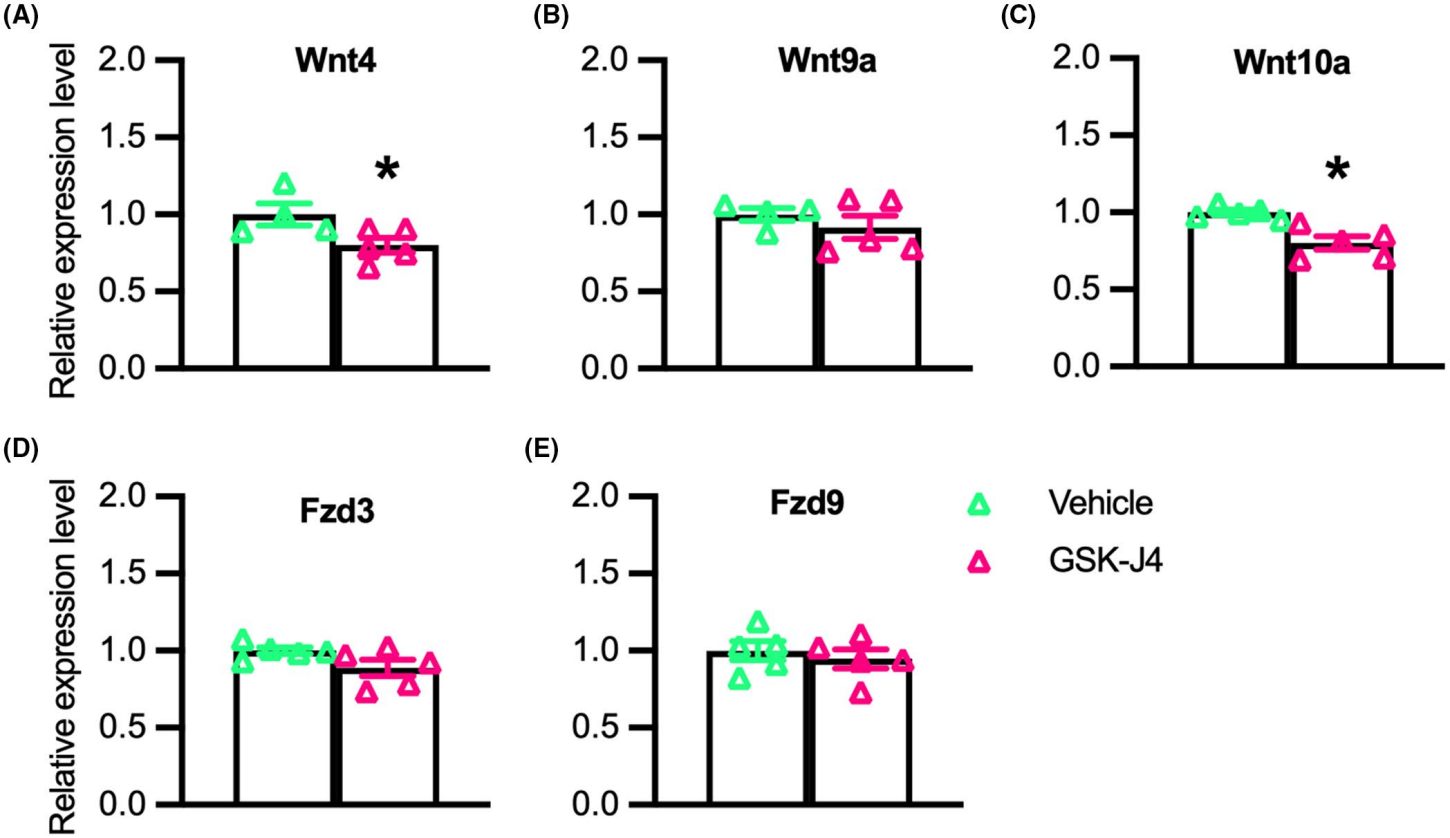
Inhibition of H3K27 demethylases reduces expression of *Wnt4* and *Wnt10a* in myogenic cells. (A–E) QPCR data showing relative expression of *Wnt4* (A), *Wnt9a* (B), *Wnt10a* (C), *Fzd*3 (D), and *Fzd9* (E) in cultured myoblasts treated with 1.2 mM of GSK‐J4 for 48‐h. *Indicates significantly different from vehicle‐treated cells at *p* < .05 analyzed by *t*‐test. Error bar represents SEM. *N* = 4 or 5 for each data set

Because our data showed that Klotho reduces *Jmjd3* expression and promotes the accumulation of H3K27 di‐ and tri‐methylation at the promoter of Wnt family members, we examined whether the reductions in Wnt transcript levels were directly related to the inhibition of H3K27 demethylase activity. QPCR data from myoblasts treated with GSK‐J4, a pharmacological inhibitor targeting H3K27 demethylases, showed that expression of *Wnt4* (Figure 6A) and *Wnt10a* (Figure 6C) were reduced by the treatment, although *Wnt9a*, *Fzd3*, and *Fzd9* (Figure 6B,D,E) were unaffected.

### 3.7 Klotho stimulation and Jmjd3 knock‐down do not have additive, inhibitory effects on the expression of Wnt4, Wnt9a, or Wnt10a

**FIGURE 7:**
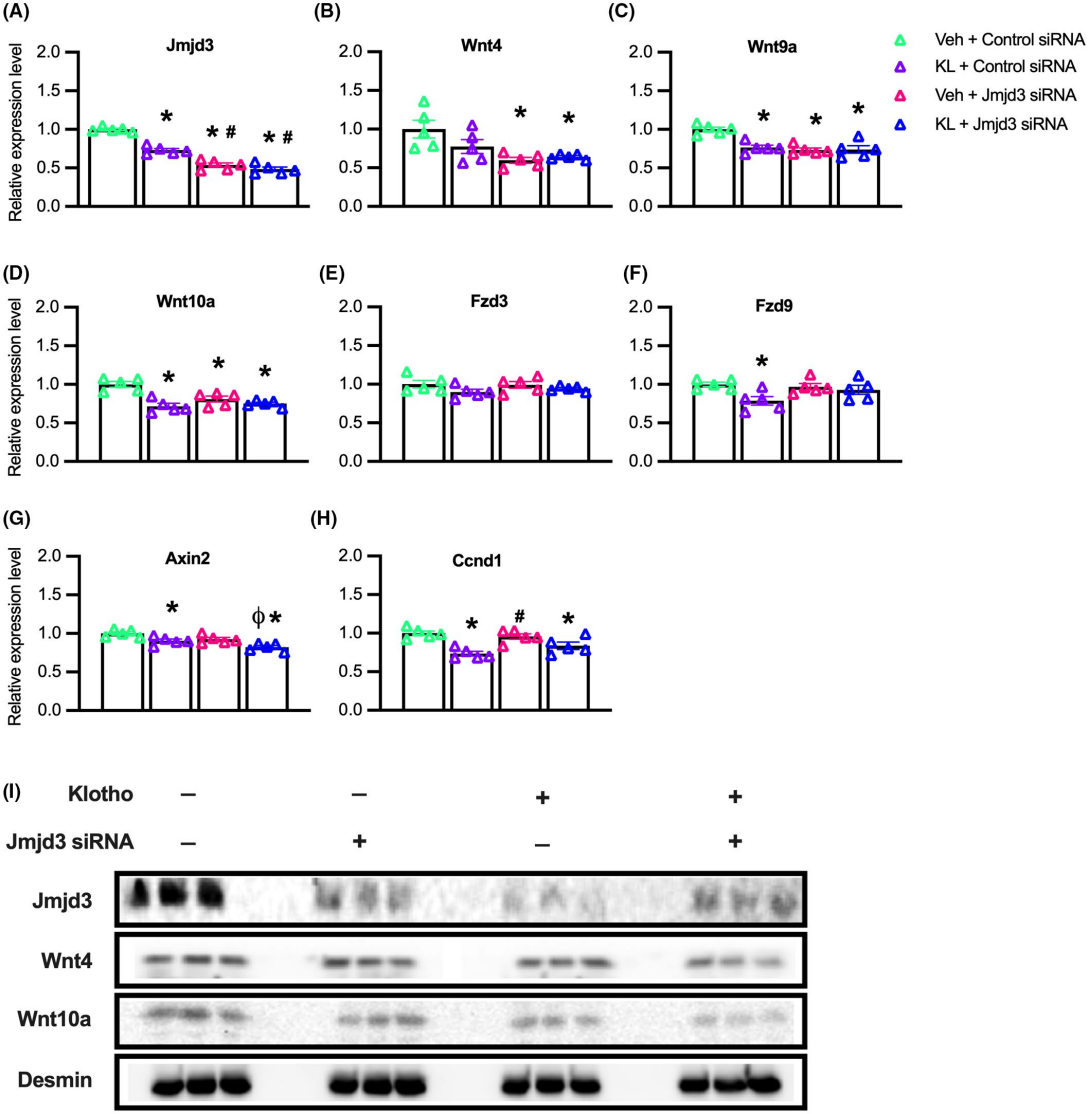
Klotho stimulation and Jmjd3 knock‐down do not have additive, inhibitory effects on the expression of *Wnt4*, *Wnt9a*, and *Wnt10a*. (A–I) Myoblasts were treated with vehicle and control siRNA (Veh + Control siRNA), Klotho and control siRNA (KL + Control siRNA), vehicle and Jmjd3 siRNA (Veh + Jmjd3 siRNA) or Klotho and Jmjd3 siRNA (KL + Jmjd3 siRNA). (A–H) QPCR data showing relative expression of *Jmjd3* (A), *Wnt4* (B), *Wnt9a* (C), *Wnt10a* (D), *Fzd3* (E), *Fzd9* (F), *Axin2* (G), and *Ccnd1* (H) in KL + Control siRNA, Veh + Jmjd3 siRNA or KL + Jmjd3 siRNA treated myoblasts compared to vehicle‐treated controls. For all bar charts, *indicates significantly different from Veh + Control siRNA treated cells at *p* < .05 analyzed by one‐way ANOVA with Tukey's multiple comparisons test. ^#^Indicates significantly different from KL + Control siRNA treated cells at *p* < .05 analyzed by one‐way ANOVA with Tukey's multiple comparisons test. ^ϕ^Indicates significantly different from Veh + Jmjd3 siRNA treated cells at *p* < .05 analyzed by one‐way ANOVA with Tukey's multiple comparisons test. Error bar represents SEM. *N* = 5 for all QPCR data sets. (I) Western blot analysis showing effects of Klotho, Jmjd3 RNAi or Jmjd3 RNAi with Klotho on Jmjd3 (180 kDa), Wnt4 (50 kDa), Wnt10a (46 kDa), and loading control desmin (60 kDa). Wnt9a protein was undetected in all groups. *N* = 3 for all groups

We next addressed whether inhibition of *Jmjd3* expression and Klotho stimulation would produce additive, inhibitory effects on the expression of Wnt family genes or Wnt target genes in myoblasts, which would indicate that they inhibited expression through separate pathways. Our qPCR data showed that treatments with recombinant Klotho and siRNA for Jmjd3 each reduced *Jmjd3* expression compared to controls, although siRNA for Jmjd3 was more effective at reducing *Jmjd3* transcripts (Figure 7A). We also found that expression of each Wnt transcript assayed (*Wnt4*, *Wnt9a*, *Wnt10a*) was significantly reduced by siRNA for *Jmjd3* but adding Klotho treatment to the inhibition with siRNA did not produce more inhibition than achieved with siRNA alone (Figure 7B–D). These findings indicate that transcriptional inhibition of these genes by blocking H3K27 demethylation through siRNA for Jmjd3 is not further enhanced by activating Klotho. Unlike the influence of siRNA for *Jmjd3* on Wnt genes, no effects on the expression of the Wnt receptors Fzd3 and Fzd9 were observed (Figure 7E,F); however, *Fzd9* expression was reduced by Klotho treatment alone (Figure 7F). Thus, Klotho may inhibit *Fzd9* through a pathway not regulated by Jmjd3. We also tested whether the reductions in Wnt ligand mRNA correlated with changes in downstream target molecules by assaying for mRNA levels of *Axin2*, a negative regulator of the Wnt pathway^58^ and *Ccnd1* which encodes cyclin D1, a positive regulator of cell cycle progression.^59^, ^60^ Similar to our findings with *Fzd9*, expression of *Axin2* and *Ccnd1* was reduced by Klotho but not by Jmjd3 siRNA (Figure 7G,H), which may indicate Klotho inhibition of these genes was independent of Jmjd3.

We next assayed whether reductions in Jmjd3 and Wnt gene expression that occurred in Klotho or Jmjd3 siRNA treated myoblasts were detectible by western blot at the protein level (Figure 7I). Both Klotho and Jmjd3 siRNA produced clear reductions in Jmjd3 protein when administered separately or combined, compared to the control group (Figure 7I). Although treatment effects on Wnt4 protein levels were less apparent (Figure 7I), Wnt10a protein was reduced by Jmjd3 siRNA treatment, by Klotho stimulation, and by the combined treatment groups compared to controls (Figure 7I). Wnt9a protein was undetectable in the control and treatment groups.

### 3.8 Klotho modulates Jmjd3 and H3K27 methylation in satellite cells

**FIGURE 8:**
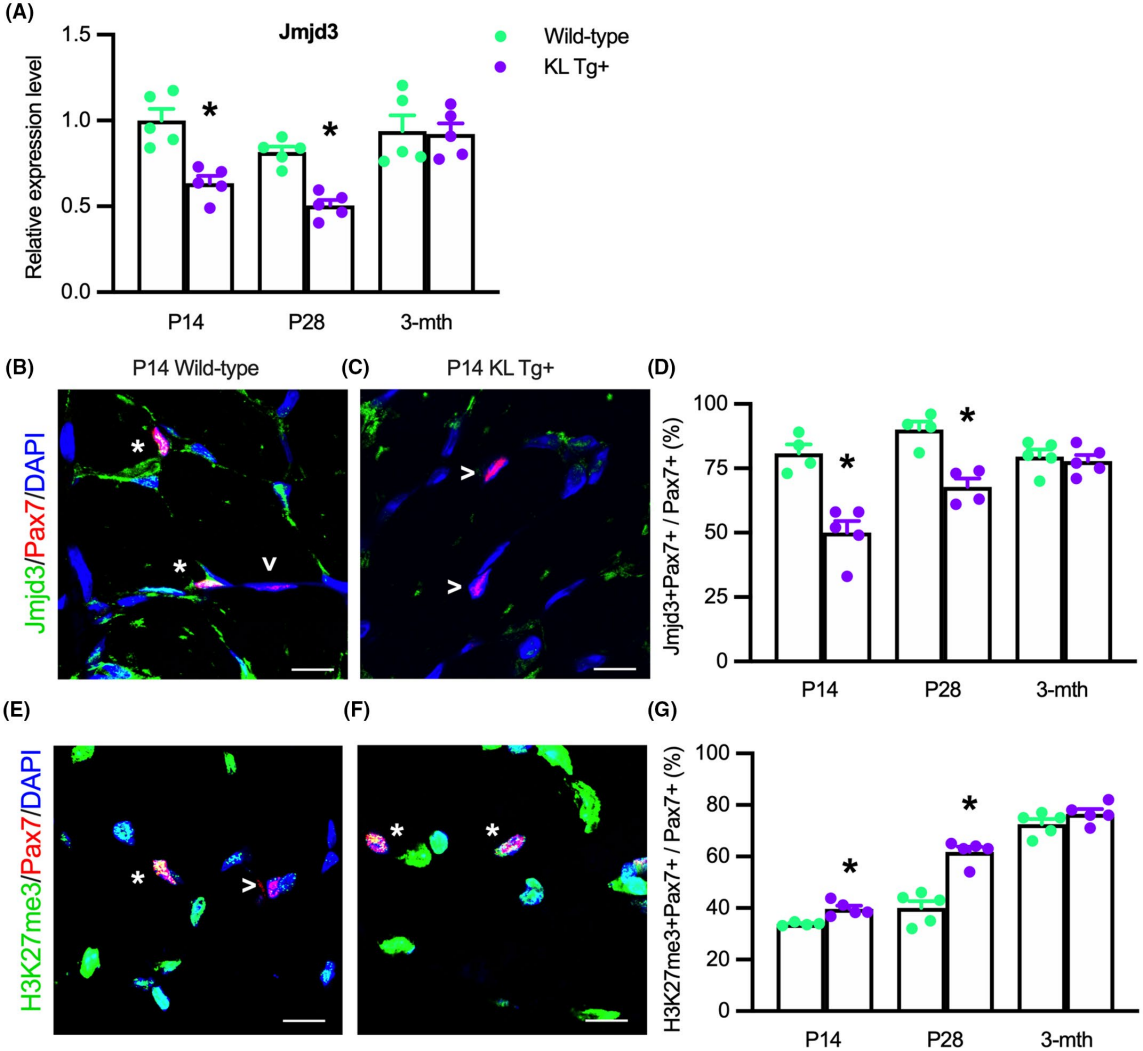
KL Tg expression reduces *Jmjd3* transcripts and localization in Pax7+ cells and increases H3K27 methylation Pax7+ cells in early postnatal development. (A) QPCR analysis showing *Jmjd3* in quadriceps muscle lysates of Wt and KL Tg+ mice. (B, C) Representative images of quadriceps muscle sections from P14 Wt (B) and KL Tg+ (C) mice showing immunofluorescent double‐labeling for Pax7 and Jmjd3. *Indicates Pax7+ cells that were also Jmjd3+. Open arrowheads (>) indicate Pax7+ single‐labeled cells. (D) The ratio of Jmjd3+/Pax7+ cells to total Pax7+ cells in sections of quadriceps muscles. (E,F) Representative images of P14 Wt (E) and KL Tg+ (F) showing immunofluorescent double labeling for Pax7 and trimethylated H3K27 (H3K27me3) in quadriceps muscle cross‐sections. *Indicates Pax7+ cells that were also H3K27me3+. Open arrowheads (>) indicate Pax7+ single‐labeled cells. (G) The ratio of H3K27me3+/Pax7+ cells to total Pax7+ cells in quadriceps muscles sections. For all bar charts, *indicates significantly different from age‐matched Wt at *p* < .05 analyzed by *t*‐test. Error bar represents SEM. *N* = 4 or 5 for each data set

We tested whether our observations that stimulation with recombinant Klotho decreased *Jmjd3* expression and elevated H3K27 methylation in myoblasts in vitro reflected the actions of Klotho during muscle development in vivo. QPCR analysis on whole quadriceps muscles shows *Jmjd3* expression is reduced at P14 and P28 but not at 3 months of age in KL Tg+ mice, compared to wild‐type mice (Figure 8A). Furthermore, we observed that Jmjd3 is present in Pax7+ cells (Figure 8B) and the proportion of Pax7+ cells expressing detectible Jmjd3 protein was reduced in KL Tg+ muscles at P14 (Figure 8C,D) and P28 (Figure 8D) but not at 3 months (Figure 8D). Next, we probed for H3K27me3 in Pax7+ cells to determine whether the reduction in Jmjd3 reflects changes in H3K27 methylation and observed that H3K27me3 was located in Pax7+ and Pax7‐ cells in developing muscle tissue (Figure 8E,F). However, the proportion of Pax7+ cells that contained detectible H3K27me3 was greater in KL Tg+ mice compared to wild‐type at P14 (Figure 8E–G) and P28 (Figure 8G) but not at 3 months (Figure 8G). We emphasize that the absence of detectible anti‐H3K27me3 binding to some satellite cell nuclei does not indicate that those cells were devoid of H3K27 methylation; the observation shows that the quantity of H2K27 marks in those cells was lower than the detection limits of the technique. These findings indicate that Klotho activation reduces Jmjd3 levels, and consequently H3K27me3, in developing muscle in vivo through 28 days.

### 3.9 Klotho reduces the expression of Wnt4, Wnt9a, and Wnt10a during early postnatal muscle growth

**FIGURE 9:**
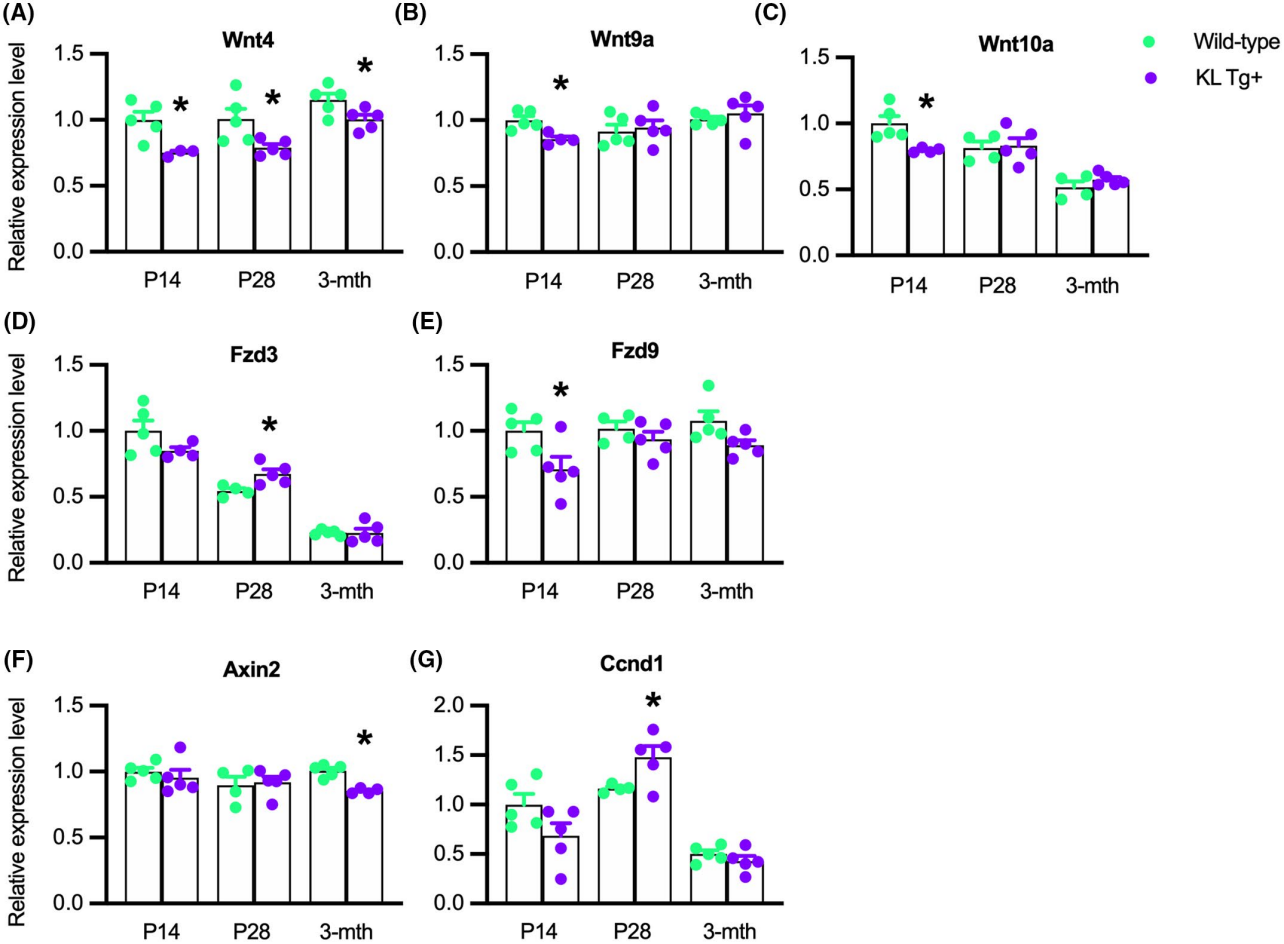
KL Tg expression reduces the expression of Wnt4, Wnt9a, and Wnt10a during early postnatal muscle growth. (A–G) QPCR data showing relative expression for transcripts of *Wnt4* (A), *Wnt9a* (B), *Wnt10a* (C), *Fzd3* (D), *Fzd9* (E), *Axin2* (F), and *Ccnd1* (G) in quadriceps muscles of Wt and KL Tg+ mice. *Indicates significantly different from age‐matched Wt at *p* < .05 analyzed by *t*‐test. Error bar represents SEM. *N* = 3–5 for each data set

Because *Jmjd3* expression was reduced in KL Tg+ muscle during early postnatal development and accompanied by elevated H3K27 methylation, we assayed for corresponding reductions in the expression of Wnt pathway genes that we found to experience increased H3K27 methylation in Klotho‐stimulated myoblasts. Similar to our in vitro findings, we found that KL Tg expression decreased expression of *Wnt4*, *Wnt9a*, and *Wnt10a* in P14 mice (Figure 9A–C). However, only *Wnt4* expression was reduced in KL Tg+ muscles at P28 or 3 months. We also observed that *Fzd9* expression was decreased at P14 resembling the effect of Klotho stimulation of myoblasts in vitro, but not affected at other ages tested (Figure 9E). Also similar to our in vitro findings, KL Tg expression did not reduce expression of *Fzd3* or *Ccnd1* (Figure 9D,E); instead the transgene produced elevations in the expression of both at P28, showing that Klotho‐driven reduction of Jmjd3 is not an important regulator of the expression of either gene. Although KL Tg expression reduced the expression of *Axin2* in muscles, the effect occurred only in 3‐month‐old mice (Figure 9F) when the transgene did not influence *Jmjd3* expression (Figure 8A), which also suggests that the transgene does not influence *Axin2* expression in muscle via Klotho suppression of Jmjd3 function, resembling our observation on myoblasts in vitro.

### 3.10 Klotho represses Wnt‐signaling in Pax7‐expressing cells during postnatal development and early adulthood

**FIGURE 10:**
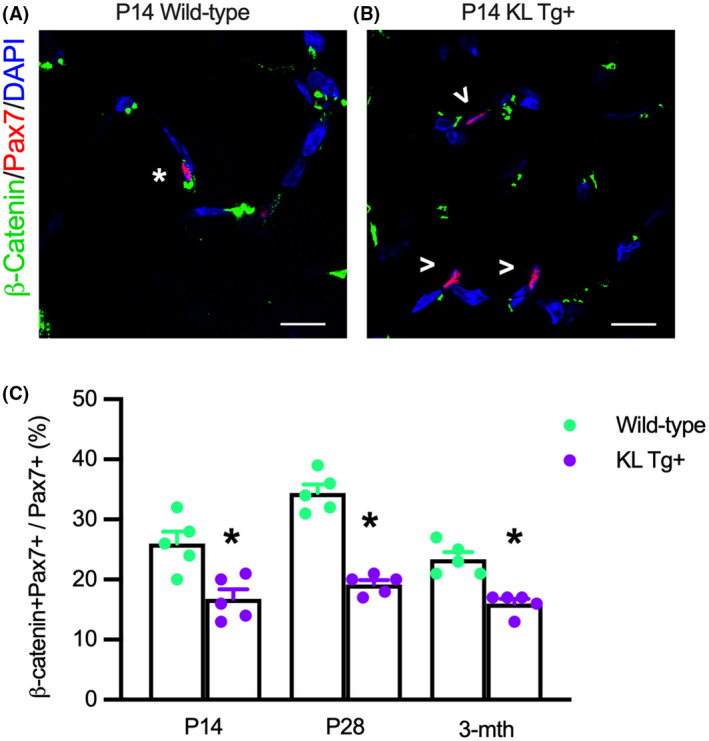
KL Tg expression reduces Wnt‐signaling in Pax7+ cells during early postnatal muscle growth. (A, B) Sections of Wt (A) and KL Tg+ (B) quadriceps muscle at P14 labeled with anti‐Pax7 (red), anti‐β‐catenin (green), and DNA labeled with DAPI (blue). *Indicates Pax7+ cells also expressing active β‐catenin+. Open arrowheads (>) indicate Pax7+ single‐labeled cells. Bar = 10 μm. (C) Ratio of Pax7+ cells that showed activated β‐catenin relative to total Pax7+ cells in Wt and KL Tg+ quadriceps muscles. *Indicates significantly different from age‐matched Wt at *p* < .05 analyzed by *t*‐test. Error bar represents SEM. *N* = 5 for each data set

Our results pertaining to the effects of Klotho on developmental myogenesis and the inhibition of the expression of Wnt ligands, Wnt receptors, and Wnt target genes suggest that muscle growth during development is influenced by fluctuating levels of Klotho. Because myogenesis is driven in part by canonical Wnt‐signaling,^54^ we assayed for activation of the Wnt‐signaling pathway in Pax7+ cells during development and in early adulthood, using an antibody to detect activated β‐catenin which medicates canonical Wnt‐signaling.^61^, ^62^ Our findings show that ~25 to 35% of Pax7+ cells expressed detectible levels of activated β‐catenin during early postnatal development in wild‐type muscle (Figure 10A,C) but the KL Tg significantly reduced the proportion of Pax7+ cells with activated β‐catenin to less than 20% (Figure 10B,C). However, we also observed that active β‐catenin in wild‐type, Pax7+ cells declines between P28 and 3 months, and elevated levels of Klotho continue to inhibit Wnt‐signaling during early adulthood (Figure 10C), indicating that Klotho influences Wnt‐signaling in myogenic cells from early postnatal development until maturity.

## 4 DISCUSSION

The function of *klotho* as an anti‐aging gene has been validated in many organs and tissues in which its age‐related loss contributes to senescence. For example, the progressive decline in Klotho in aging skeletal muscle diminishes mitochondrial function in myogenic cells and reduces the regenerative capacity of muscle.^63^ In addition, the accelerated, epigenetic silencing of *klotho* expression in dystrophic muscle contributes to losses of muscle function, reductions in satellite cell numbers, and increases in muscle fibrosis, all of which are characteristics of aging muscle.^12^ Because of those associations between reductions of *klotho* expression in aging and diseased muscle and physiological changes associated with aging, we were surprised to learn that the period of most rapid reduction of *klotho* expression occurs in the first few weeks of postnatal muscle development,^12^ suggesting that Klotho may play a significant, regulatory role in development, as well as aging. The findings of our investigation show that increases in *klotho* expression during postnatal muscle growth cause transient increases in satellite cell numbers and affect the rate of muscle fiber growth in young mice. Furthermore, our results identify a novel pathway through which Klotho can influence myogenesis by reducing expression of the histone demethylase Jmjd3 in muscle, leading to reductions in the expression of Wnt family genes and inhibition of canonical Wnt signaling in satellite cells.

The transient increase in Pax7+ cells in the postnatal muscle that was caused by expression of the KL Tg indicates that Klotho stimulates the expansion of populations of activated myogenic cells, but does not influence satellite cell activation. Furthermore, those increases in numbers of myogenic cells are attributable to increased proliferation because Klotho stimulation of activated myogenic cells increases the proportion that contains nuclear Ki67, a marker of cell proliferation, without affecting apoptosis or necrosis.^12^ At P14, when over 80% of satellite cells are activated,^4^, ^5^ we found that elevated Klotho production caused the greatest expansion of Pax7+ cell numbers. However, our data show that the transgene had no effect on numbers of Pax7+ cells at 3 months of age when fewer than 1% of satellite cells are in the cell cycle.^7^ The amplification of satellite cell numbers during the first 3 weeks of postnatal development can have long‐term consequences on muscle growth because the majority of those cells fuse with existing fibers to become myonuclei and the adult number of myonuclei is established by P21.^9^ Although the Klotho‐mediated amplification of Pax7+ cell proliferation in early postnatal development was short‐lived, we found that muscle fibers in Klotho transgenic mice were over 24% larger in diameter than fibers in wild‐type muscles at 3 months of age, which corresponds to ~24 years of age for humans. This long‐term increase in muscle fiber size that extends into adulthood is converse to the consequence of ablating satellite cells from early postnatal muscle. Experimental depletion of ~70% of satellite cells from P28 mouse muscles significantly reduced the subsequent growth of muscle fibers.^10^ These observations indicate that the developmental significance of the relatively high levels of Klotho expression that occur in muscles of early postnatal mice is to amplify the numbers of activated myogenic cells, which then increase muscle growth at subsequent stages of development. They also show that the transient delivery of exogenous factors to growing muscles during early postnatal growth could lead to larger muscle fibers in adulthood.

Because of the well‐established importance of epigenetic regulatory factors for controlling the proliferation and differentiation of myogenic cells, we assayed whether the influence of Klotho on myogenesis could be mediated by changes in the expression of enzymes that are involved in epigenetic modification of myogenic genes. Although Klotho did not affect the expression of some of the best‐characterized epigenetic regulatory factors involved in myogenesis, we observed a strong downregulation of Jmjd3 in myoblasts stimulated with Klotho in vitro and in muscles expressing the KL Tg in vivo. Jmjd3 plays a significant role in removing silencing histone marks from genes that regulate development from the earliest stages of embryogenesis through to differentiation of specific cell lineages in adult organisms. In the early mesodermal lineage, from which skeletal muscle eventually arises, Jmjd3 influences mesoderm differentiation, and Jmjd3 mutation in embryonic stem cells increase H3K27 methylation at the promoter of the mesodermal regulator, *Brachyury*, leading to reductions in Wnt‐induced mesodermal differentiation.^64^ Although a role for Jmjd3 in affecting myogenesis has not been identified in previous investigations, the forced expression of ectopic Jmjd3 in human pluripotent stem cells can induce their expression of muscle‐specific genes, including Pax7.^30^ That observation suggested the possibility that Jmjd3 may also regulate the development of committed myogenic cells, which our data now verify. Notably, the downregulation of *Jmjd3* expression and the reduced proportion of Pax7+ cells that expressed detectible Jmjd3 in Klotho transgenic muscles occurred in young muscles, but not in adult muscles. This indicates that the regulatory roles of Klotho modulation of Jmjd3 may be complementary to the role of another H3K27 demethylase, UTX, in adult myogenesis. Although no defects in developmental myogenesis were observed in mice in which *Utx* was ablated in satellite cells, myogenesis in adult muscle following acute injury was impaired in the mutants, leading to slower muscle growth and regeneration following injury.^29^

Our findings that Klotho reduced the expression of *Wnt* genes in muscle in vivo and in vitro and that the inhibitory effects on *Wnt4*, *Wnt9a*, *Wnt10a*, and *Fzd9* expression generally declined as postnatal development proceeded, indicates that the effects of Klotho on early postnatal myogenesis occur, in part, through inhibition of Wnt signaling. The reduced expression of *Wnt* genes specifically in early postnatal development is important because signaling initiated by Wnt binding to receptors in the Fzd family has powerful influences on myogenesis. For example, signaling through the canonical, β‐catenin‐dependent Wnt pathway is required for satellite cell differentiation^54^ and pharmacological activation of the canonical pathway enhances muscle differentiation.^54^, ^65^, ^66^, ^67^ Wnt4, Wnt9a, and Wnt10a can increase β‐catenin activity leading to activation of the canonical pathway.^68^, ^69^, ^70^, ^71^, ^72^, ^73^ Similarly, Wnt ligation of Fzd9 can increase activation of the canonical pathway.^74^, ^75^, ^76^ Numerous observations support the conclusion that Wnt4, Wnt9a, and Wnt10a can promote muscle differentiation. The expression of each is elevated at the onset of muscle differentiation, coinciding with increases in β‐catenin activation^56^, ^57^ and overexpression of Wnt4 in differentiating muscles increased expression of target genes in the canonical pathway.^57^ In addition, overexpression of either Wnt4 or Wnt9a increased muscle differentiation in vitro.^56^, ^57^ Although inhibition of Wnt signaling by Klotho could also potentially occur through Jmjd3‐independent mechanisms that have not been identified, our findings show that the primary pathway activated by Klotho for inhibition of at least some Wnt family members involves Jmjd3. We found that the magnitude of inhibition of expression of Wnt4, Wnt9a, and Wnt10a in Klotho‐treated cells was not further increased by Jmjd3 siRNA treatments, indicating that reductions in the expression of those Wnt family members by Klotho and Jmjd3 siRNA occurred predominantly through a common pathway.

The negative regulation of the expression of Wnt family members by Klotho introduces a novel, epigenetic mechanism through which Klotho can influence Wnt function and myogenesis. Previous investigators have shown that Klotho can bind to Wnt1, Wnt3a, Wnt4, Wnt5a, and Wnt7a^77^, ^78^ and have shown that the binding can inhibit the activity of at least Wnt3a in a cell‐free system.^77^ Furthermore, Klotho treatment of isolated muscle fibers in vitro diminished Wnt signaling, which was attributed to Klotho binding to extracellular Wnt.^22^ However, our findings show that Klotho can influence Wnt function and myogenesis through an epigenetic pathway. There are important, physiological differences between Wnt inhibition by binding soluble Klotho in the extracellular space versus the novel mechanism we propose. First, Wnt inhibition achieved by maintaining gene silencing of Wnt family members would provide a mechanism for long‐term inhibition that does not require continuous secretion of Klotho. In addition, the mechanism that we propose would suppress the expression of specific Wnt receptors only in cells that express Klotho receptors. This would provide more specific targeting of the inhibitory influence than achieved by Klotho acting only as an extracellular Wnt antagonist.

Although our findings show that Klotho decreases the expression of Wnt family members in myogenic cells, which is associated with increases in myogenic cell proliferation and reductions in their differentiation, there may be other less direct pathways through which increases in Klotho influence Wnt‐mediated regulation of myogenesis that we have not identified in this investigation. For example, because Wnt4 is a secreted ligand that can act through autocrine or paracrine pathways, there may be non‐muscle cell types in vivo in which Wnt4 expression is reduced by Klotho, leading to less activation of the canonical Wnt pathway in muscle cells through a paracrine effect. Nevertheless, as shown by previous investigators,^56^ knock‐down of Wnt4 expression in myoblasts is sufficient to significantly reduce their differentiation, expression of myogenin, and their subsequent growth as myotubes, following fusion. Thus, the reduction of Wnt expression in myogenic cells that are stimulated with Klotho or in which Jmjd3 expression is reduced is sufficient to explain the reductions in myogenin expression, muscle differentiation, and fiber growth that we report in our investigation.

The most parsimonious interpretation of our findings in light of current knowledge of the role of Wnt signaling in muscle differentiation is that Klotho acts on myogenic cells after their activation, leading to inhibition of Wnt expression and diminished signaling through the canonical Wnt pathway. That disruption in Wnt signaling slows myogenic cell differentiation which produces a transient amplification of myogenic cell numbers. In addition to expanding numbers of myogenic cells by delaying their differentiation, the pro‐mitotic influence of Klotho on activated myogenic cells would further increase their numbers.^12^ In natural, postnatal myogenesis this regulatory influence of Klotho would be limited, in part, by the decline in Klotho production in young mice as development proceeds. However, as our findings show when reductions in *klotho* expression in muscle are prevented by the expression of a KL Tg, the influences of Klotho on the numbers of Pax7+ cells and the level of expression of Jmjd3 and specific members of the Wnt family still occur only in early postnatal myogenesis. That observation shows that additional, unidentified mechanisms are in place that limits the influences of Klotho in early postnatal muscle development, in addition to changes in *klotho* expression. Those mechanisms are subject to continuing studies.

## DISCLOSURES

The authors declare no competing or financial interests.

## AUTHOR CONTRIBUTIONS

Conceptualization: Cynthia M. McKee, Michelle Wehling‐Henricks, James G. Tidball; Methodology: Cynthia M. McKee, Douglas J. Chapski, Manuel Rosa‐Garrido, Michelle Wehling‐Henricks, James G. Tidball; Software: Douglas J. Chapski; Investigation: Cynthia M. McKee, Michelle Wehling‐Henricks, James G. Tidball; Resources: Douglas J. Chapski, Makoto Kuro‐o, Manuel Rosa‐Garrido, Thomas M. Vondriska, James G. Tidball; Writing–original draft: Cynthia M. McKee, James G. Tidball; Review, editing, and approval of final draft: Cynthia M. McKee, Douglas J. Chapski, Michelle Wehling‐Henricks, Makoto Kuro‐o, Manuel Rosa‐Garrido, Thomas M. Vondriska, James G. Tidball; Funding acquisition: Cynthia M. McKee, Thomas M. Vondriska, James G. Tidball.

## ETHICAL APPROVAL

All experiments involving the use of animals were performed according to the National Institutes of Health Guide for the Care and Use of Laboratory Animals. All protocols were approved by the Chancellor's Animal Research Committee at the University of California, Los Angeles (Animal Welfare Assurance number A‐3196).

## Supporting information

Fig S1

Supplementary Material

## Data Availability

All data that were analyzed and contributed to this investigation are included in this published article or in Supplemental Information in the online version of this article.
