# Supplementary figures and images for: The anti‐aging protein Klotho affects early postnatal myogenesis by downregulating Jmjd3 and the canonical Wnt pathway
Source: FASEB J. 2022 Feb 17;36(3):e22192. doi: 10.1096/fj.202101298R (PMC9007106; doi:10.1096/fj.202101298R)

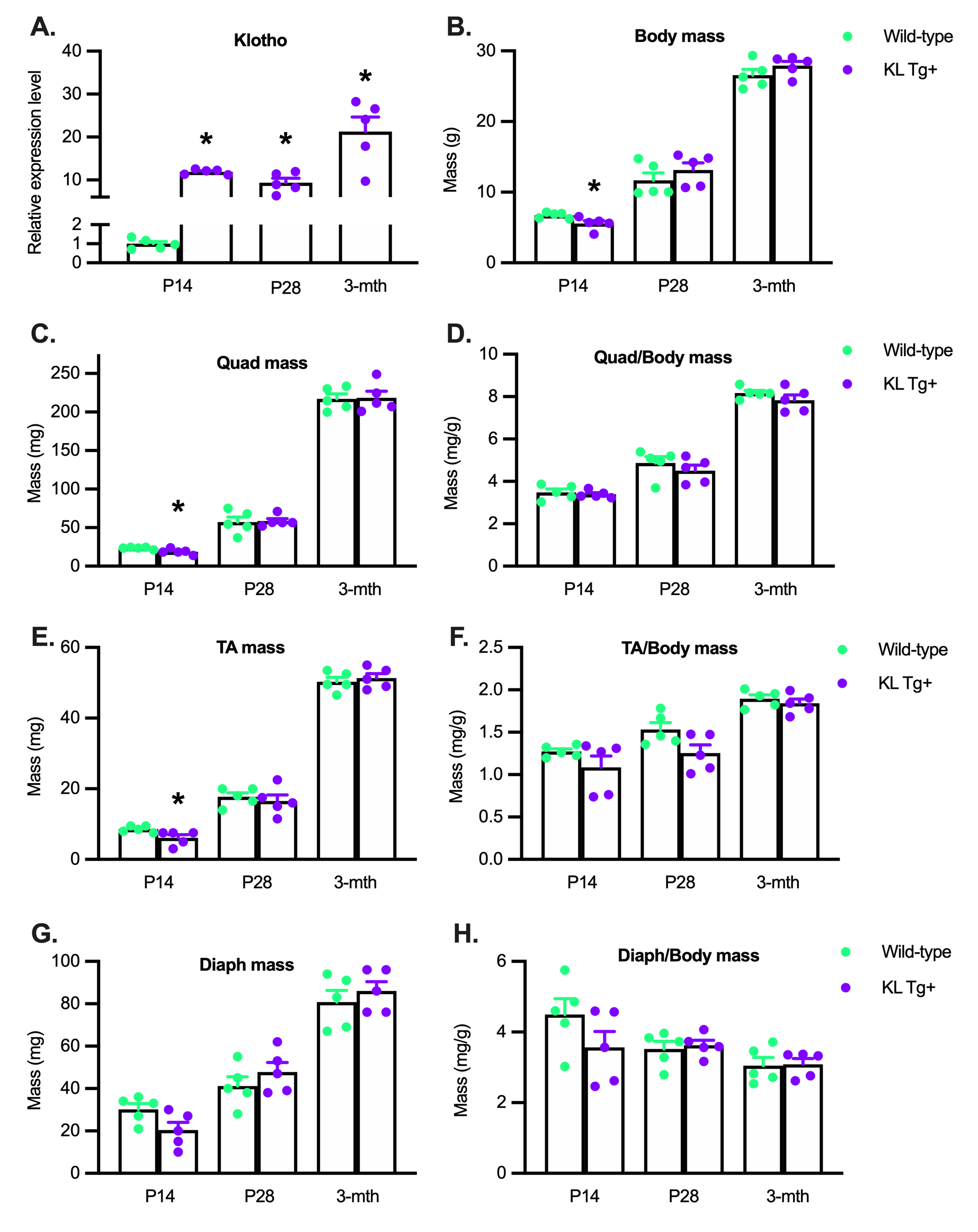

Supplement: Supplementary file 1 — Fig S1 [file FSB2-36-0-s002.tif]
