# Supplementary material for: The anti‐aging protein Klotho affects early postnatal myogenesis by downregulating Jmjd3 and the canonical Wnt pathway
Source: FASEB J. 2022 Feb 17;36(3):e22192. doi: 10.1096/fj.202101298R (PMC9007106; doi:10.1096/fj.202101298R)
Supplement: Supplementary file 2 — Supplementary Material [file FSB2-36-0-s001.docx]

Supplemental Figure 1. Expression of a *klotho* transgene affects body mass and hindlimb muscle mass. (A) QPCR data showing relative expression of *klotho* in quadriceps muscle lysates of P14 Wt and P14, P28 and 3-months KL Tg+ mice show expression of a *klotho* transgene increases *klotho* transcripts throughout development. * indicates significantly different from P14 Wt at *p* < 0.05 analyzed by one-way ANOVA followed by Dunnett’s multiple comparisons test. Error bar represents SEM. N = 5 for each data set. (B) Body mass (g) of Wt and KL Tg+ mice at P14, P28 and 3-months. (C) Quadriceps mass (mg) of Wt and KL Tg+ mice. (D) Quadriceps mass normalized to body mass (mg/g) of Wt and KL Tg+ mice. (E) TA mass (mg) of Wt and KL Tg+ mice. (F) TA mass normalized to body mass (mg/g) of Wt and KL Tg+ mice. (G) Diaphragm mass (mg) of Wt and KL Tg+ mice. (H) Diaphragm mass normalized to body mass (mg/g) of Wt and KL Tg+ mice. For B-H, * indicates significantly different from age-matched Wt at *p* < 0.05 analyzed by *t*-test. Error bar represents SEM. N = 5 for each data set.
